# Metabolism of coclaurine into the WADA-banned substance higenamine: a doping-relevant analytical evaluation of Kampo extracts

**DOI:** 10.1007/s11418-025-01940-4

**Published:** 2025-08-02

**Authors:** Seiichi Sakamoto, Kouta Osaki, Hiroko Abe, Yorie Tayama, Akito Tsuruta, Poomraphie Nuntawong, Satoru Koyanagi, Varalee Yodsurang, Satoshi Morimoto

**Affiliations:** 1 Department of Pharmacognosy, Graduate School of Pharmaceutical Sciences, Kyushu University, 3-1-1 Maidashi, Higashi-Ku, Fukuoka 812-8582 Japan; 2 Biodesign Inc, 3-25-15 Nishi Ikebukuro, Toshima, Tokyo 171-0021 Japan; 3 Department of Pharmaceutics, Graduate School of Pharmaceutical Sciences, Kyushu University, 3-1-1 Maidashi, Higashi-Ku, Fukuoka 812-8582 Japan; 4 Department of Pharmacology and Physiology, Faculty of Pharmaceutical Sciences, Chulalongkorn University, Bangkok, 10330 Thailand; 5 Center of Excellence in Preclinical Toxicity and Efficacy Assessment of Medicines and Chemicals, Chulalongkorn University, Bangkok, 10330 Thailand

**Keywords:** Higenamine, Coclaurine, Doping, Kampo medicines, Crude drugs, Screening

## Abstract

Higenamine, a β2-agonist, has been listed as a prohibited substance by the World Anti-Doping Agency (WADA) since 2017, poses a doping risk through the use of traditional herbal formulations. In Japan, Kampo medicines, composed of multiple crude drugs, are widely used, raising concerns about the unintentional intake of banned substances. In this study, urinary excretion of higenamine was observed in mice following coclaurine administration, and higenamine formation was confirmed in human liver microsomes, indicating a potential risk associated with coclaurine-containing herbs. Therefore, 128 Kampo extract products were analyzed to identify crude drugs containing higenamine and/or coclaurine using lateral flow immunoassay (LFA), enzyme-linked immunosorbent assay (ELISA), and liquid chromatography–tandem mass spectrometry (LC–MS/MS) analysis. Consequently, fourteen crude drugs were identified to contain higenamine and/or coclaurine. Notably, six crude drugs—including Magnolia bark, Japanese Zanthoxylum peel, Jujube seed, Magnolia flower, Cimicifuga rhizome, and Coptis rhizome—were newly confirmed to contain higenamine, while nine—including Cinnamon bark, Magnolia bark, Euodia fruit, Asiasarum root, Japanese Zanthoxylum peel, Cimicifuga rhizome, Jujube, Processed Aconite root, and Phellodendron bark—were newly identified as containing coclaurine. These results underscore the potential risk of doping violations associated with coclaurine, which may be metabolized into higenamine, although coclaurine is not currently classified as a prohibited substance. Our findings highlight the need for regulatory consideration to mitigate unintentional doping risks among athletes using Kampo medicine.

**Graphical abstract:**

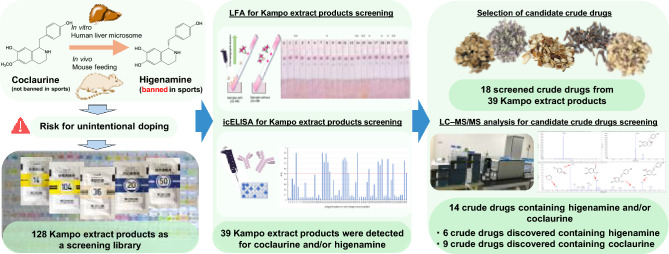

**Supplementary Information:**

The online version contains supplementary material available at 10.1007/s11418-025-01940-4.

## Introduction

**Fig. 1:**
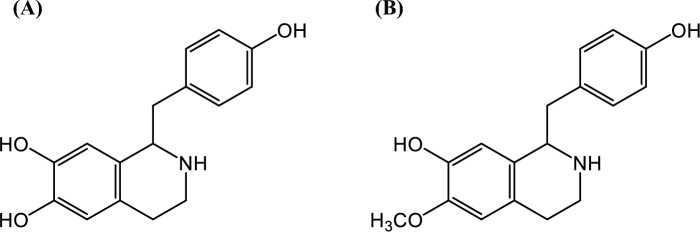
Structures of **A** higenamine and **B** coclaurine

Higenamine (Fig. 1A, norcoclaurine, demethylcoclaurine) is a benzylisoquinoline alkaloid initially identified in the root of *Aconitum japonicum* Thunberg (Ranunculaceae) [1], after which it was found in *Asarum heterotropoides* F. Maekawa var. *mandshuricum* F. Maekawa (Aristolochiaceae) [2], *Nelumbo nucifera* Gaertner (Nelumbonaceae) [3], *Nandina domestica* Thunberg (Berberidaceae) [4], *Tinospora crispa* (L.) Miers ex Hook.f. & Thomson (Menispermaceae) [5], *Gnetum parvifolium* (Warb.) W.C. Cheng (Gnetaceae) [6], *Sinomenium acutum* Rehder et Wilson (Menispermaceae) [7], and *Phellodendron* spp. (Rutaceae) [8]. The biosynthesis of higenamine has revealed that dopamine and 4-hydroxyphenylacetaldehyde were stereospecifically condensed by norcoclaurine synthase to form a specific enantiomer, (*S*)-higenamine [9, 10]. Higenamine has recently garnered significant attention due to its various pharmaceutical activities, including antianalgesic [11], anti-inflammatory [11], antioxidative [12], antidepressant [13], and antitumor effects [14]. In 2017, higenamine was included in the International Standard Prohibited List of the World Anti-Doping Agency (WADA) as an all-time banned substance under the S3 group β2-agonist due to its function as a partial agonist of the β-adrenergic receptor [15]. Due to its β2-agonistic effects, higenamine increases the cardiac output and subsequent heart rate, resulting in improved athletic performance [16]. In addition, Jing et al. investigated the anabolic activity of higenamine and its mechanisms of action in vitro using C2C12 myotubes. They found that higenamine induces an anabolic effect in C2C12 cells, evidenced by increased myotube diameter, gene expression, and the induction of MHC protein expression [17]. According to the WADA guidelines, an adverse analytical finding (AAF) is considered when a concentration of free higenamine in human urine exceeds 10 ng/mL. Recently, crude drugs containing higenamine have also garnered significant attention to prevent unintentional doping, as over 90% of medical doctors in Japan have experienced prescribing Kampo medicines consisting of various crude drugs.

Coclaurine (Fig. 1 B, 6-*O*-methylhigenamine) is produced from (*S*)-higenamine by 6-*O*-methyltransferase as (*S*)-enantiomer in a plant [18]. Despite its structural similarity to higenamine, it is not classified as a banned substance by WADA. The metabolites of higenamine in mammals include coclaurine and its isomer isococlaurine (7-*O*-methylcoclaurine), both of which have been identified in rat urine following oral administration of higenamine, using gas chromatography–mass spectrometry (GC–MS) [19]. Higenamine and coclaurine have also been detected in human urine after the administration of throat lozenges containing *Nandina domestica*, known to contain higenamine, using ultra-performance liquid chromatography–tandem mass spectrometry (UPLC–MS/MS) [4, 20]. Recently, it has been reported that methylated, sulfated, and glucuronidated higenamine were detected as the metabolites of higenamine in human urine by quadrupole-orbitrap LC–MS/MS [21]. Intriguingly, Shang et al. reported that higenamine was conversely detected in rat urine and feces samples following the oral administration of crude drug extract from the dried mature seed of *Ziziphus jujuba* Mill. var. *spinosa* Hu ex H.F. Chou, containing a high amount of coclaurine [22, 23]. This suggests that coclaurine may be metabolized to higenamine in vivo. In other words, this suggests that the intake of coclaurine, which is not a banned substance, could lead to unintentional doping. Therefore, the metabolism of coclaurine is of significant interest from an anti-doping perspective.

In this study, therefore, we primarily investigated the metabolites of coclaurine after the oral administration of coclaurine to mice and elucidated that the free and glucuronide forms of higenamine could be detected in urine samples by liquid chromatography with tandem mass spectrometry (LC–MS/MS) analysis. Furthermore, they were also detected when coclaurine was treated with pooled human liver microsomes (HLM). These results raise the possibility of a doping violation due to the intake of the Kampo medicines and crude drugs containing coclaurine. Thus, we used 128 kinds of Kampo extract products as a compound library to identify crude drugs containing higenamine and/or coclaurine using immunological techniques, including lateral flow immunoassay (LFA) [24] and enzyme-linked immunosorbent assay (ELISA) [25]. These methods, previously developed with a monoclonal antibody against higenamine (MAb E8), were found to also recognize coclaurine. Eighteen crude drugs were selected from 39 Kampo extract products using immunological techniques, and subsequent LC–MS/MS analysis revealed that 14 of these crude drugs contained higenamine and/or coclaurine, among which several food ingredients were included. As a result, six crude drugs—Magnolia bark, Japanese Zanthoxylum peel, Jujube seed, Magnolia flower, Cimicifuga rhizome, and Coptis rhizome—were newly identified to contain higenamine, while nine crude drugs—Cinnamon bark, Magnolia bark, Euodia fruit, Asiasarum root, Japanese Zanthoxylum peel, Cimicifuga rhizome, Jujube, Processed Aconite root, and Phellodendron bark—were newly identified to contain coclaurine. Since the aforementioned crude drugs are used in various Kampo medicines and food ingredients commonly used, this study provides valuable information for athletes who rely on Kampo medicines as well as those who take these herbs in their daily life.

This paper demonstrates the metabolites of coclaurine in mice and pooled HLM, immunological screening from Kampo extract products, and the verification of crude drugs containing higenamine and/or coclaurine by LC–MS/MS analysis.

## Materials and methods

### Materials and reagents

(*S*)-Higenamine hydrobromide (98%) and (*S*)-coclaurine hydrochloride (98%) were purchased from Toronto Research Chemicals Inc. (Toronto, Canada). Human microsomes (lot PL050H-A, mixed gender pool from 50 donors) were purchased from Gibco (Thermo Fisher Scientific, Waltham, MA, USA). Alamethicin and uridine 5′-diphosphoglucuronic acid (UDPGA: U6751) were purchased from Cayman Chemical Co. (Ann Arbor, MI, USA) and Sigma-Aldrich (St. Louis, MO, USA), respectively. A total of 128 Kampo extract products and Processed Aconite root were obtained from Tsumura & Co. (Tokyo, Japan), where the indicated numbers correspond to the product No. of TSUMURA. To verify the presence of higenamine and coclaurine, 18 kinds of screened crude drugs were obtained from Uchida Wakanyaku Co., Ltd. (Tokyo, Japan), Tochimoto Tenkaido Co., Ltd. (Osaka, Japan), and Kojima Kampo Co., Ltd. (Osaka, Japan). Diazepam-d5, provided as the NAGINATA Internal Standard Solution, was purchased from Hayashi Pure Chemical Ind., Ltd. (Osaka, Japan). Goat anti-mouse IgG pAb (HRP) (ab6789) and Rabbit anti-mouse IgG pAb (ab6709) for the ELISA and LFA, respectively, were purchased from Abcam (Cambridge, MA, USA). All other reagents were of analytical grade.

### Metabolites of coclaurine in mice

Animal experiments were conducted at UNITECH Co., Ltd. (Chiba, Japan) following approval from the animal ethics committee (Approval Number: AGR KDI-221025A-27 at UNITECH Co., Ltd.). Three male BALB/cCrSlc mice, aged 6 weeks, were obtained from Japan SLC Inc. (Shizuoka, Japan). During the rearing period, they were provided with ad libitum access to standard chow (CE-2, CLEA Japan, Inc., Tokyo, Japan) and water, and kept under a 12 h light/dark cycle at 22 °C–26 °C. For the coclaurine solution, distilled water (2 mL) was added to coclaurine (1 mg) and sonicated for 10 min to prepare the coclaurine solution. After a 12 h fasting period before drug administration, coclaurine solution (0.5 mg/mL, 15 mg/kg, average weight; 20.7 g) was administered once by forced oral gavage. The mice were transferred to a metabolic gauge, and urine and feces were collected at 12, 24, and 48 h after administration.

Sample preparation for LC–MS/MS analysis is detailed in the Supplementary Materials.

### Metabolites of coclaurine in HLM

Metabolism of coclaurine in pooled HLM was investigated by incubating coclaurine with pooled HLM at 37 °C. The stock solution of coclaurine was prepared in methanol, with the final methanol concentration in the solution being 0.03% (v/v). The metabolism of coclaurine (1.0 μM) was performed using pooled HLM (0.5 mg/mL) and that with UDPGA (5 mM) for phase I and II metabolisms, respectively, in 100 mM potassium phosphate buffer (pH 7.4, 100 µL) containing 2.0 mM MgCl_2_. The reactions were initiated by adding a cofactor solution containing 1.3 mM NADPH. At time 0, 1, 3, 6, 12, and 24 h, the reactions were terminated by addition of acetonitrile containing diazepam-d5 at 50 ng/mL (100 µL). The samples were centrifuged at 12,000 rpm for 5 min at 25 °C. The supernatant was collected and used as the sample for LC–MS/MS analysis. The samples were stored at 4°C until further use.

### LC–MS/MS analysis

An ExionLC™ LC System (SCIEX, Framingham, MA, USA) equipped with a ZORBAX Eclipse Plus C18 Rapid Resolution HT column (2.1 mm × 100 mm, 1.8 µm; Agilent Technologies, Santa Clara, CA, USA) or a CORTECS T3 column (2.1 mm × 100 mm, 2.7 µm; Waters, Milford, MA, USA), along with an X500R QTOF System (SCIEX, Framingham, MA, USA) was used. The injection volume was 3 μL. The mobile phase consisted of 0.1% (v/v) formic acid (solvent A) and acetonitrile (solvent B) delivered at a constant flow rate of 0.25 mL/min. The column temperature was set at 40 °C, and the gradient profile was applied as described in Table S1. The MS data were acquired in the positive ionization mode using information-dependent acquisition, an artificial intelligence-based product ion scan mode. Each sample was measured three times. The MS and MS/MS spectra were analyzed using SCIEX OS software (SCIEX, Framingham, MA, USA).

### Screening of Kampo extract products by LFA using MAb E8

The Kampo extract products consisting of higenamine- and coclaurine-containing crude drugs were initially screened from 128 Kampo extract products by LFA, previously developed using an MAb E8 [24].

In short, the test strip of LFA was composed of three parts, which are the sample pad, nitrocellulose membrane, and adsorbent pad. Cellulose fiber-based paper, cut into 1.5 × 0.6 cm strips, was used for the sample and adsorbent pads without any treatment. The nitrocellulose backing (Hi-Flow Plus 240, Millipore, Temecula, CA, USA), cut into 5.4 × 0.6 cm strips, mainly functioned as the LFA. On the nitrocellulose membrane, control and test zones were prepared by immobilizing Rabbit anti-mouse IgG pAb antibodies (1 mg/mL, 0.5 μL) and higenamine–γ-globulin conjugates (2 mg/mL, 1.5 μL), respectively. After the membrane was dried, its surface was blocked by soaking in 1% (w/v) bovine serum albumin in phosphate-buffered saline (PBS) for 2 h at room temperature. The membrane was washed three times with PBS containing 0.05% (v/v) Tween-20 (PBS-t), followed by drying at room temperature. The test strip was assembled by affixing a sample pad to the bottom of the dried membrane and an adsorption pad to the top.

For screening of the Kampo extract products consisting of higenamine- and coclaurine-containing crude drugs, 20-fold diluted samples of the Kampo extract products in 5% (v/v) methanol (20 µL) were mixed with the detection reagent containing colloidal gold conjugated-MAb E8 (130 µL) and allowed to stand for 5 min before the test strip was dipped into the mixed solution for 15 min for detection. The results were interpreted by visual observation. Since the assay was based on a competitive system, the absence of pink color relative to the control sample of 5% (v/v) methanol was considered positive (+ + or +), while the presence of pink color matching intensity of the control sample was interpreted as negative (−).

### Screening of Kampo extract products by indirect competitive ELISA (icELISA) using MAb E8

The Kampo extract products consisting of higenamine- and coclaurine-containing crude drugs were subsequently screened from 128 Kampo extract products by icELISA previously developed using an MAb E8 [25].

In brief, higenamine-ovalbumin (OVA) conjugates (2 μg/mL) in 50 mM carbonate buffer (pH 9.6, 100 μL/well) were incubated for 1 h at 37 °C to immobilize the conjugates onto the surface of an immunoplate (Nunc, Maxisorb, Roskilde, Denmark). After washing the plate, PBS containing 5% (w/v) skimmed milk (PBS-sm, 300 μL/well) was added to each well and incubated for 1 h at 37 °C to prevent nonspecific adsorption of other proteins. Subsequently, various concentrations of (*S*)-higenamine (9.77−625 ng/mL) or 20-fold diluted samples of Kampo extract products in 5% (v/v) methanol (50 μL/well) were incubated with MAb E8 (100 ng/mL, 50 μL/well) for 1 h at 37 °C. The plate was then incubated with a 16,000-fold diluted solution of Goat anti-mouse IgG pAb (HRP)(100 μL/well) for 1 h at 37 °C to detect MAb E8 binding to the immobilized higenamine-OVA conjugates. Finally, the plate was treated with 2,2′-azino-bis(3-ethylbenzothiazoline-6-sulfonic acid) diammonium salt (0.3 mg/mL) in 0.1 M citrate buffer (pH 4.0), supplemented with 0.003% (v/v) H_2_O_2_ (100 μL/well) for 20 min at 37 °C to develop color. The absorbance at 405 nm was subsequently measured using a Multiskan™ microplate reader. PBS-t was used to wash the plate thrice between each step.

In the screening by icELISA, the ratio of absorbance at 405 nm from the Kampo extract product samples (*A*) to that of 5% (v/v) methanol used as a negative control (*A*_*0*_), denoted as *A*/*A*_*0*_, was used for evaluation. Values of *A*/*A*_*0*_ < 0.5 were considered positive (+), while values of *A*/*A*_*0*_ ≥ 0.5 were considered negative (−).

The cross-reactivities (CRs) of MAb E8 against (*S*)-coclaurine were calculated using the following equation [26]:

$${\mathrm{CR}}\text{s (\%) }=\frac{{\mathrm{IC}}{50}\text{ for }{\mathrm{(S)}}\mathrm{-higenamine}}{{\mathrm{IC}}{50}\text{ for }{\mathrm{(S)}}\mathrm{-coclaurine}} \times 100$$

## Results

### Metabolites of coclaurine in mice

To investigate the metabolites of coclaurine in mice, urine and feces were collected at 12, 24, and 48 h after the oral administration of authentic coclaurine to BALB/cCrSlc mice (0.5 mg/mL, 15 mg/kg). These samples were analyzed by LC–MS/MS using a ZORBAX Eclipse Plus C18 Rapid Resolution HT column with a gradient system (Table S1(A)), with higenamine, coclaurine, and their glucuronide conjugates as the target compounds for detection. The monoisotopic ion peak corresponding to authentic higenamine ([C_16_H_17_NO_3_ + H]^+^ at *m*/*z* 272.1281) was produced at *m*/*z* 272.1282 with a retention time (Rt) of ~ 8.6 min. Associated fragment ion peaks were observed at *m*/*z* 255.1024 ([C_16_H_15_O_3_]^+^), 161.0604 ([C_10_H_9_O_2_]^+^), and 107.0499 ([C_7_H_7_O]^+^) (Fig. S1(A)). Similarly, the monoisotopic peak corresponding to authentic coclaurine ([C_17_H_19_NO_3_ + H]^+^ at *m*/*z* 286.1438) was observed at *m*/*z* 286.1432 with a Rt of ~ 9.4 min, with fragment ions detected at *m*/*z* 269.1176 ([C_17_H_17_O_3_]^+^), 175.0761 ([C_11_H_11_O_2_]^+^), and 107.0497 ([C_7_H_7_O]^+^) (Fig. S1(B)).

**Fig. 2:**
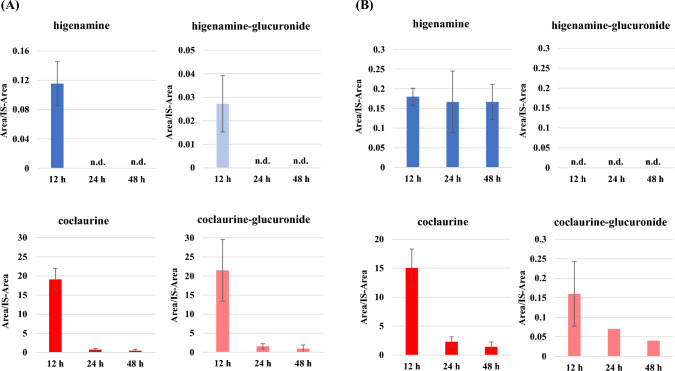
Detection of higenamine and coclaurine in mice **A** urine and **B** feces by LC–MS/MS analysis. The samples were collected at 12, 24, and 48 h after oral administration of authentic coclaurine into BALB/cCrSlc (0.5 mg/mL, 15 mg/kg). Area and IS-area indicate area of sample and internal standard (diazepam-d5), respectively. n.d.: not detected

In the urine samples, two metabolites showed *m*/*z* values of 448.1602 (C_22_H_25_NO_9_) and 462.1759 (C_23_H_27_NO_9_) as the [M + H]^+^ ion at Rt of ~ 7.5 and ~ 8.0 min, respectively. These metabolites also exhibited fragment ion peaks corresponding to higenamine and coclaurine, respectively (Fig. S2), suggesting the presence of glucuronide conjugates of higenamine (higenamine-glucuronide) and those of coclaurine (coclaurine-glucuronide) as metabolites of coclaurine. Interestingly, in addition to free coclaurine, free higenamine (60.0 ng/mL; data not shown) was detected in the urine sample at 12 h (Fig. 2A).

On the other hand, the free forms of higenamine, coclaurine, and coclaurine-glucuronide were detected in the fecal samples, with coclaurine showing the highest amounts (Fig. 2B).

### Metabolites of coclaurine in HLM

**Fig. 3:**
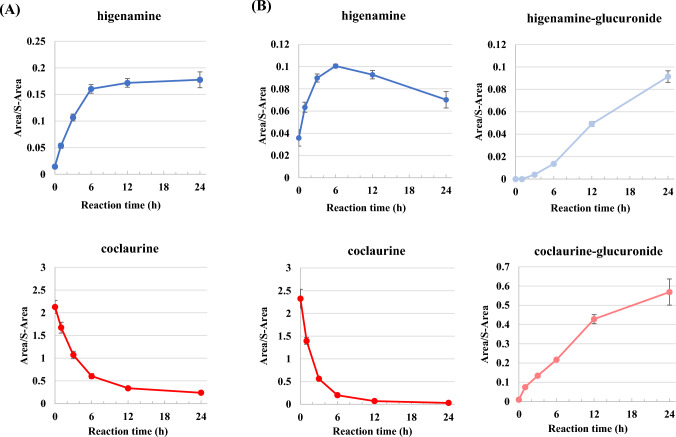
In vitro metabolisms of coclaurine in **A** phase I and **B** phase II reaction by HLM. Coclaurine was incubated with HLM, and metabolites at 0, 1, 3, 6, 12, and 24 h were analyzed by LC–MS/MS based on higenamine, coclaurine, and their glucuronide conjugates. Area and S-Area indicate area of sample and internal standard (diazepam-d5), respectively

To investigate the metabolites of coclaurine in HLM, coclaurine was incubated with HLM, and metabolites were analyzed at 0, 1, 3, 6, 12, and 24 h using LC–MS/MS, focusing on higenamine, coclaurine, and their glucuronide conjugates. In the phase I metabolism (Fig. 3A), it was observed that as the amount of coclaurine decreased over time, the relative amount of higenamine increased. In the phase II metabolism (Fig. 3B), similar to phase I, the amount of coclaurine decreased over time. However, the rate of coclaurine decline in phase II metabolism was approximately twice as fast as in phase I metabolism as its glucuronide conjugates in addition to higenamine, along with its glucuronide, were produced [27]. Focusing on time-dependent metabolites, the amount of higenamine increased, reaching a maximum at 6 h, and then gradually decreased. Accordingly, higenamine-glucuronide dramatically increased after 6 h.

### CRs of MAb E8

The CRs of the anti-higenamine monoclonal antibody (MAb E8) against (*S*)-coclaurine were evaluated to determine whether MAb E8 could be used as a tool in an immunoassay for the screening of crude drugs containing both higenamine and coclaurine from Kampo extract products, as the intake of coclaurine may potentially cause unintentional doping. Upon calculation of the CRs of MAb E8 by icELISA, it was found to exhibit a 45.8% CRs. This result suggested that MAb E8 can be applied to LFA and icELISA for screening crude drugs containing higenamine and/or coclaurine.

### Screening of Kampo extract products by LFA and icELISA using MAb E8

**Fig. 4:**
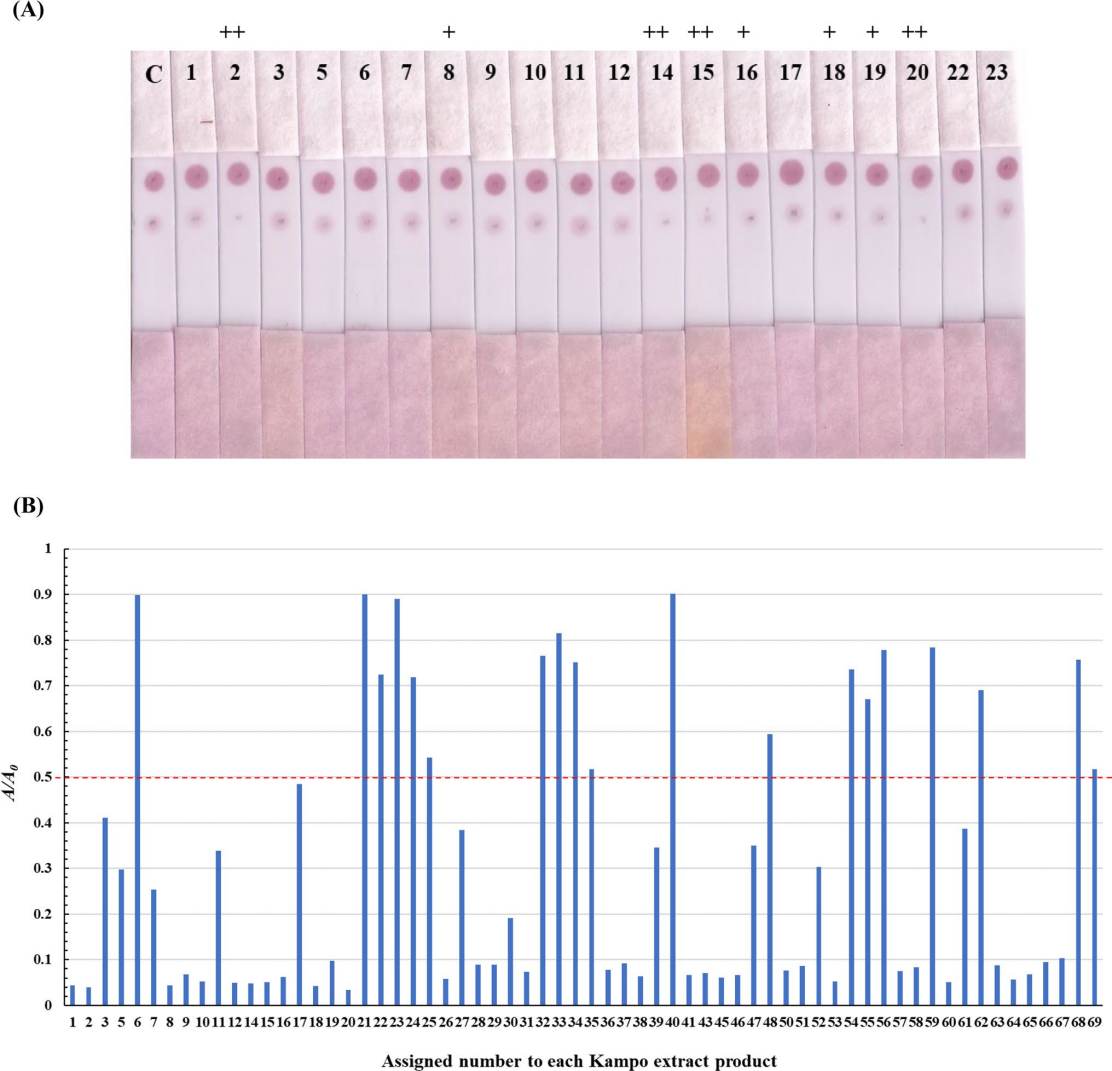
Screening of crude drugs containing higenamine and/or coclaurine from 128 kinds of Kampo extract products by **A** LFA and (B) icELISA using MAb E8. (A) C indicates the control, where 5% (v/v) methanol was used, and the numbers [1–23 (except 21)] on the strip test correspond to the product No. of TSUMURA assigned to 128 Kampo extract products. The (+ +) and (+) indicate positive and weak positive, respectively. The results using the remaining Kampo medicines are provided as Fig. S3. (B) *A* and *A*_*0*_ indicate the absorbance obtained from each sample of Kampo extract products and the negative control (5% (v/v) methanol), respectively. The numbers (1–69) of the X axis correspond to the product No. of TSUMURA assigned to 128 Kampo extract products. The results using the remaining Kampo medicines are provided as Fig. S4

Kampo medicines, which consist of various kinds of crude drugs rich in plant secondary metabolites, can serve as a valuable compound library for screening potential targets of interest. In this study, we collected 128 kinds of Kampo extract products that are clinically used in Japan and screened them for crude drugs that produce higenamine and/or coclaurine. Since LFA is a simple, rapid, and specific assay based on the high affinity between antigen and antibody, the target compound can be detected without the need for complicated pretreatment, even when the sample contains various matrices [28]. Therefore, LFA is an appropriate tool for the preliminary screening of higenamine- and/or coclaurine-containing crude drugs from various Kampo extract products. Primarily, LFA using MAb E8 was applied to all prepared samples, with the limit of detection of the LFA determined to be 3.13 µg/mL [24]. In this screening by LFA, no spots and pale spots observed on the test zone were classified as positive (+ +) and weak positive (+), respectively. As a result, 39 Kampo extract products exhibited positive results (+ + and +) (Figs. 4A and S3).

Subsequently, icELISA was applied to all 128 Kampo extract product samples, as icELISA has also been reported to be a specific and sensitive assay suitable for plant samples [29]. In the screening by icELISA, *A*/*A*_*0*_ was evaluated for screening, where *A*/*A*_*0*_ < 0.5 and *A*/*A*_*0*_ ≥ 0.5 were classified as positive (+) and negative (−), respectively. The icELISA screening revealed that 90 Kampo extract products were positive (Figs. 4B and S4). These results indicated that all 39 Kampo extract products screened by LFA were found to be positive in icELISA, as summarized in Table 1.

**Table 1:**
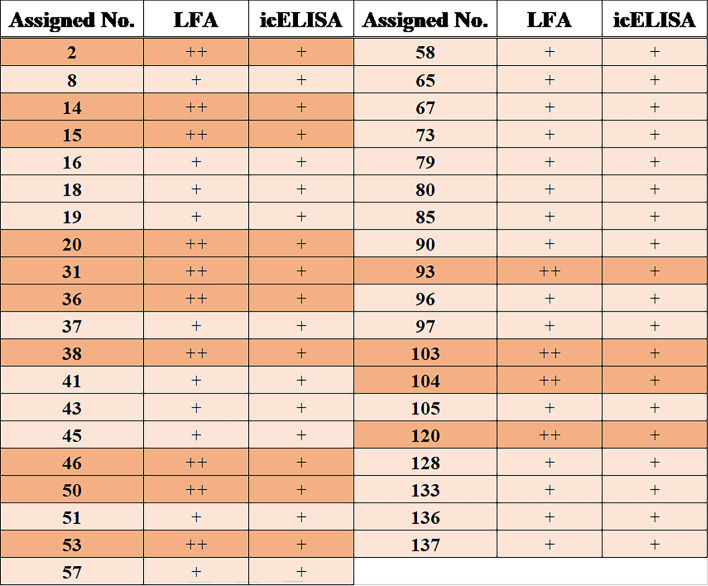
Summary of the screening of crude drugs containing higenamine and/or coclaurine from 128 kinds of Kampo extract products by LFA [24] and icELISA [25] using MAb E8.

The indicated numbers correspond to the product No. of TSUMURA.

Based on the screening results by LFA and icELISA, the biosynthesis pathway of higenamine and coclaurine, and a comprehensive literature review, the crude drugs that were considered to contain higenamine and/or coclaurine were narrowed down. Based on the screening results, the component crude drugs of the Kampo formulation that exhibited negative results were excluded from the candidates, as they either did not contain or contained trace amounts of the target compounds. In addition, among the component crude drugs of the Kampo formulation that showed positive, those known to contain the target compounds, as well as those from the same genus, were considered candidates. Crude drugs containing berberine and magnoflorine were also considered, as they have been reported to be produced through the same biosynthesis pathway as higenamine and coclaurine [30]. The results suggested that 18 crude drugs were screened and identified as containing higenamine and/or coclaurine, as shown in Table 2.

**Table 2:** Candidates of the crude drugs containing higenamine and/or coclaurine.^a^

Crude drugs reported to contain higenamine
Cinnamon bark [36] Euodia fruit [37] Asiasarum root [2] Clove [38] Processed Aconite root [1] Sinomenium stem and rhizome [7] Nelumbo seed [3]
Crude drugs reported to contain coclaurine
Jujube seed [39] Magnolia flower [40] Nelumbo seed [3] Sinomenium stem and rhizome [7]
Crude drugs reported to contain berberine or magnoflorine
[berberine] Phellodendron bark [41] Coptis rhizome [41] [magnoflorine] Magnolia bark [42] Japanese Zanthoxylum peel [43] Jujube seed [44] Jujube [42] Sinomenium stem and rhizome [42] Phellodendron bark [42] Coptis rhizome [41]
Crude drugs belonging to the same genus as those containing higenamine ^b^
Japanese Zanthoxylum peel Cimicifuga rhizome
Crude drugs belonging to the same genus as those containing coclaurine
Magnolia bark Jujube
Crude drugs belonging to the same genus as those containing berberine or magnoflorine ^c^
[berberine] Alpinia officinarum rhizome [magnoflorine] Magnolia flower
Crude drugs that have been suggested to possibly contain higenamine [45]
Immature orange Citrus unshiu peel

^a^Their definition of each crude drug is summarized in Table S2

^b^Cimicifuga rhizome and Japanese Zanthoxylum peel are crude drugs in the same genus as black cohosh (*Cimicifuga racemosa* (L.) Nutt) [46] and prickly pepper (*Zanthoxylum bungeanum* Maxim) [36], respectively, which have been reported to contain higenamine

^c^Alpinia officinarum rhizome is a crude drug in the same genus as *Alpinia galanga*, which has been reported to contain berberine [47]

### Detection of higenamine and coclaurine in candidate crude drugs by LC–MS/MS analysis

The 18 selected crude drugs were purchased from two different companies and used in the experiment to ensure the reliability of the results. They are summarized along with their definition in Table S2. In this study, Group A was defined as the group of crude drugs purchased mainly from Tochimoto Tenkaido Co., Ltd., except for Processed Aconite root obtained from Tsumura & Co. Group B was defined as the group of crude drugs purchased mainly from Uchida Wakanyaku Co., Ltd. except for Japanese Zanthoxylum peel obtained from Kojima Kampo Co., Ltd (Table S3).

The crude drugs provided in non-powder form were ground using an electric mill, and all were extracted using methanol. The extracts were prepared as a 20% (v/v) methanol solution and used for the detection of the target compounds, higenamine, and coclaurine, by LC–MS/MS. A total of 36 extracts were analyzed by LC–MS/MS using either a ZORBAX Eclipse Plus C18 Rapid Resolution HT column or a CORTECS T3 column. As a result, the monoisotopic ion peaks corresponding to higenamine ([C_16_H_17_NO_3_ + H]^+^ at *m*/*z* 272.1281) were detected in 13 crude drugs (Table S3) at Rt of ~ 5.4 and ~ 5.1 min using the gradient system of (B) and (C), respectively (Table S1), as well as the fragment ion peaks for higenamine ([C_16_H_15_O_3_]^+^ at *m*/*z* 255.1016, [C_16_H_13_O_2_]^+^ at *m*/*z* 237.0910, [C_10_H_9_O_2_]^+^ at *m*/*z* 161.0597, and [C_7_H_7_O]^+^ at *m*/*z* 107.0491) (Fig. S5(A)). On the other hand, monoisotopic ion peaks corresponding to coclaurine ([C_17_H_19_NO_3_ + H]^+^ at *m*/*z* 286.1438) were observed in 13 crude drugs (Table S3) at Rt of ~ 6.0 and ~ 5.7 min using the gradient system of (B) and (C), respectively (Table S1). In addition, the MS/MS spectrum showed fragment ion peaks for coclaurine ([C_17_H_17_O_3_]^+^ at *m*/*z* 269.1172, [C_16_H_13_O_2_]^+^ at *m*/*z* 237.0910, [C_11_H_11_O_2_]^+^ at *m*/*z* 175.0754, and [C_7_H_7_O]^+^ at *m*/*z* 107.0491) (Fig. S5(B)).

### Determination of higenamine and coclaurine in candidate crude drugs by LC–MS/MS analysis

**Fig. 5:**
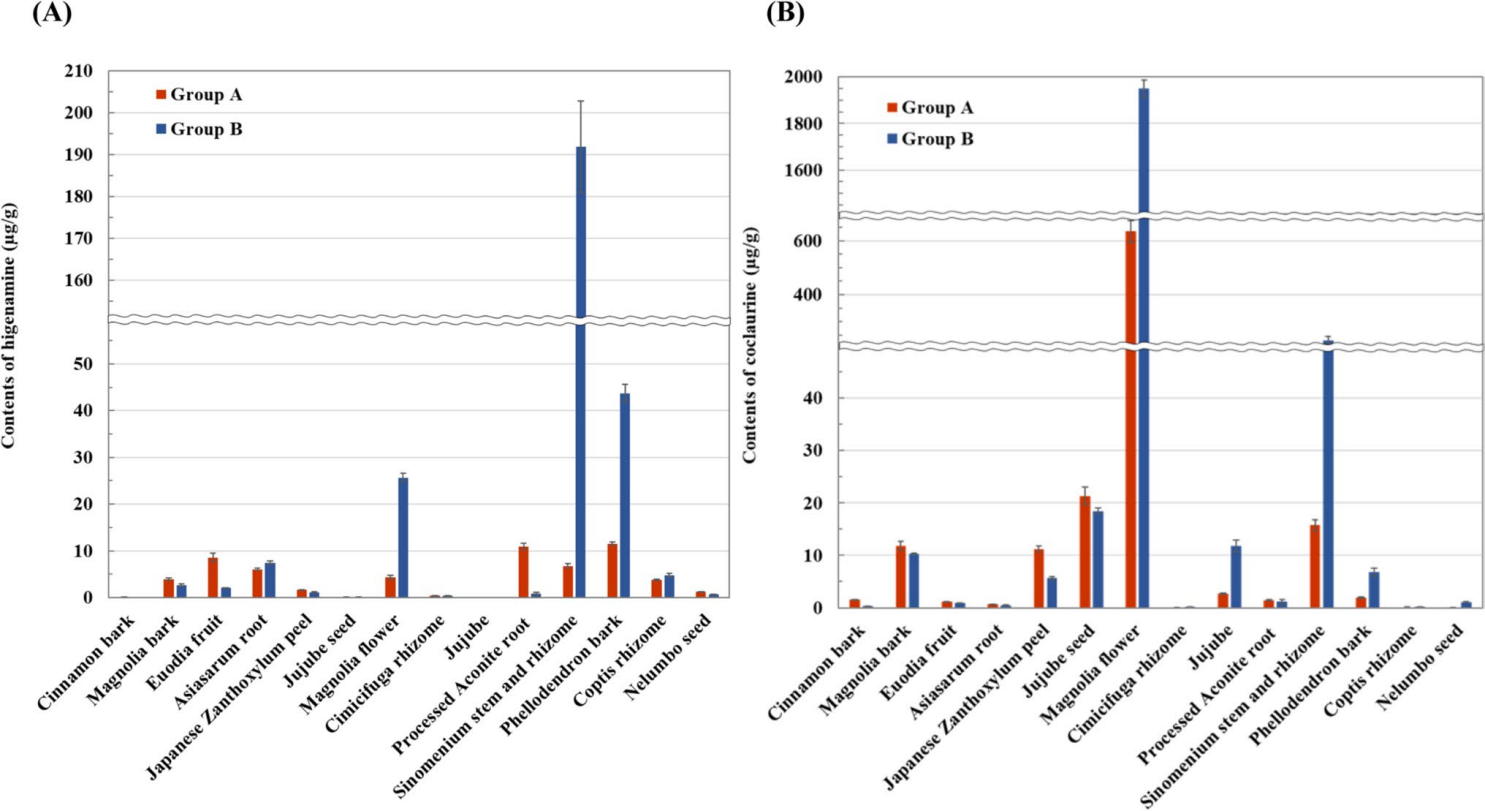
Determination of (A) higenamine and (B) coclaurine in the 14 crude drugs by LC–MS/MS analysis. Quantitative results were calculated as the content of higenamine and coclaurine (μg) per gram of powder in each crude drug

Calibration curves for higenamine and coclaurine were constructed using seven concentrations (1, 5, 10, 25, 50, 100, and 250 ng/mL) within the range of 1–250 ng/mL for their determination in 14 crude drugs (28 samples in total), excluding four crude drugs (Immature orange, Clove, Citrus unshiu peel, and Alpinia officinarum rhizome) in which neither higenamine nor coclaurine was detected. Quantitative results were calculated as the content of higenamine and coclaurine (μg) per gram of powder in each crude drug (Fig. 5). In this quantitative analysis, the area of XIC at *m/z* 272.128 ± 0.005 and 286.144 ± 0.005 was used for the calculation of the content of higenamine and coclaurine, respectively. As a result, higenamine was determined in 26 samples, excluding Jujube, where no higenamine was detected, while coclaurine was determined in all 28 samples.

## Discussion

Higenamine, an anti-doping substance banned by WADA, was detected in rat urine and feces following the oral administration of a crude drug extract from the dried mature seeds of *the Ziziphus jujuba* Mill. var. *spinosa* Hu ex H.F. Chou, which is known to contain a high amount of coclaurine [22, 23]. This result suggests that the intake of coclaurine could potentially lead to unintentional doping. Therefore, the metabolism of coclaurine must be addressed. Urine and feces were collected at 12, 24, and 48 h after the oral administration of authentic coclaurine to BALB/cCrSlc mice.

In the urine samples, higenamine- and coclaurine-glucuronides were identified as metabolites of coclaurine. Interestingly, free higenamine was also detected in the urine sample, indicating that coclaurine is metabolized into higenamine in vivo. When the relative amount of metabolites was examined by comparing the peak ratio with that of the internal standard, the relative amounts of higenamine and its glucuronides were found to be small compared with those of coclaurine and its glucuronides (Fig. 2A). However, considering the species and individual differences in drug metabolism, the intake of coclaurine, which is not a banned substance, can cause doping violations. When focusing on the time after oral administration, the highest amount of urinary metabolites was observed at 12 h in the following order: coclaurine ≈ coclaurine-glucuronide > higenamine > higenamine-glucuronide. This rapid urinary excretion of the benzylisoquinoline alkaloid coclaurine aligned with the pattern observed for higenamine metabolites following oral administration in humans, where the highest concentration of free higenamine was detected in urine samples at 6 and 9 h post-administration [21]. These results strongly suggested the possibility that higenamine can be detected following the oral administration of coclaurine-containing substances.

Similarly, the free forms of higenamine, coclaurine, and coclaurine-glucuronide were detected in the fecal samples (Fig. 2B). In a rat study, oral administration of berberine, a benzylisoquinoline alkaloid like coclaurine, resulted in most metabolites being excreted in the feces as free berberine [31]. This aligned with our findings, where the free coclaurine was predominantly detected. The detection of free higenamine in feces further confirmed that coclaurine was metabolized into higenamine in vivo.

To investigate the activity of hepatic drug-metabolizing enzymes in vitro using human cell-based hepatic drug-metabolizing enzyme, pooled HLM were used, in which cytochrome P450 (CYP) and UDP-glucuronosyltransferases (UGTs) are responsible for phase I and phase II metabolisms, respectively (Fig. 3). Coclaurine was incubated with HLM, and metabolites were analyzed over time using LC–MS/MS. The phase I metabolism indicated that *O*-demethylation occurred to form higenamine during this phase [32–34]. In the phase II metabolism, higenamine- and coclaurine-glucuronides were newly detected in addition to higenamine. These results in phase II metabolism suggested that coclaurine was primarily metabolized to higenamine and coclaurine-glucuronide by CYP and UGTs, respectively, followed by the formation of higenamine-glucuronide from its free form by UGTs [21]. The metabolism of coclaurine observed using HLM also supported the findings from the mice studies, showing that coclaurine was metabolized into higenamine. These findings raise questions about the validity of listing only higenamine in WADA’s International Standard Prohibited List [35].

In our previous study, MAb E8 exhibited low levels of CRs against natural benzylisoquinoline alkaloids, including berberine and papaverine, with CRs of 3.48% and < 0.06%, respectively [25]. Since MAb E8 was newly found to possess CRs against coclaurine with 45.8%, MAb E8 can be applied to LFA and icELISA for screening crude drugs containing higenamine and/or coclaurine. LFA, followed by icELISA using MAb E8 was applied to 128 Kampo extract product samples. Thirty-nine of these samples were screened as positive (Table 1), and the crude drugs potentially containing higenamine and/or coclaurine were subsequently narrowed down based on the analysis of their constituent crude drugs, the known biosynthesis pathways of higenamine and coclaurine, and a comprehensive literature review. As shown in Table 2, a total of 18 crude drugs were identified through screening as a potential source of higenamine and/or coclaurine, and were subsequently subjected to further analysis using LC–MS/MS.

The LC–MS/MS analysis revealed that 14 crude drugs contained higenamine and/or coclaurine (Table S3). Among the identified crude drugs, Cinnamon bark, Japanese Zanthoxylum peel, Jujube, and Nelumbo seed are commonly used as food ingredients, particularly in spices and desserts. Notably, six crude drugs—Magnolia bark, Japanese Zanthoxylum peel, Jujube seed, Magnolia flower, Cimicifuga rhizome, and Coptis rhizome—were newly identified as containing higenamine. In addition, nine crude drugs—Cinnamon bark, Magnolia bark, Euodia fruit, Asiasarum root, Japanese Zanthoxylum peel, Cimicifuga rhizome, Jujube, Processed Aconite root, and Phellodendron bark—were newly found to contain coclaurine. Among them, some were already reported to contain other benzylisoquinoline alkaloids. Coptis rhizome and Phellodendron bark contain palmatine and magnoflorine, in addition to berberine [41, 42]. In addition, Magnolia bark, Japanese Zanthoxylum peel, Jujube seed, and Jujube are known to contain magnoflorine [42–44]. This suggests that higenamine and coclaurine may be present in plants containing benzylisoquinoline alkaloids (e.g., morphine, codeine, ipecac, berberine, magnoflorine, and palmatine). Considering that higenamine and coclaurine are intermediates in the biosynthesis pathway of benzylisoquinoline alkaloids, this study provided valuable information on the Cimicifuga rhizome, which may contain useful benzylisoquinoline alkaloids.

Conversely, for four crude drugs (Immature orange, Clove, Citrus unshiu peel, and Alpinia officinarum rhizome), in which neither higenamine nor coclaurine was detected, Clove is a crude drug that has been described in textbooks and online sources as containing higenamine. Furthermore, in the “2017 Prohibited List International Standard on Higenamine”, Clove is also listed as a crude drug containing higenamine by the Japan Anti-Doping Agency (JADA) [48]. However, no reference data supporting the presence of higenamine in Clove have been reported so far, except for an abstract from a symposium in 1978 [38]. In this study, higenamine was not detected in Clove. This supported the report claiming that higenamine was not detected in Clove by LC–MS/MS [8] and in any parts of the original plant of Clove by LC–TOF/MS [49].

Considering the biosynthesis pathway of coclaurine, its presence in the plant suggests that higenamine may also be present [18]. In this study, only coclaurine was detected in Jujube (*Zizyphus jujuba* Mill. var. *inermis* Rehder) excluding seed, although the original plant of Jujube belongs to the same genus as that of the Jujube seed (*Zizyphus jujuba* Mill. var. *spinosa* Hu ex H.F. Chou), in which both higenamine and coclaurine were detected. This result suggested that higenamine may be present in the seeds of the original Jujube plants. Although a direct comparison between Jujube and Jujube seed is not feasible due to differences in botanical variety, the seeds were found to contain a slightly higher amount of coclaurine than the fruit (Fig. 5B).

The content of higenamine and coclaurine in the crude drugs (26 samples for higenamine and 28 samples for coclaurine) was determined by LC–MS/MS analysis, as shown in Fig. 5. The target compound in each crude drug varied by up to 20-fold or more between the groups, although there was no difference in the presence or absence of the target compound between groups. These differences can be attributed to the nature of crude drugs as natural products, which are influenced by a range of factors, such as geographical location, environmental factors (such as soil and weather), temporal factors (such as harvest year and season) genetic variations of the original plants, and cultivation and processing conditions [50]. Among the crude drugs, the Magnolia flower and Sinomenium stem and rhizome contained high amounts of both higenamine and coclaurine, while Phellodendron bark and Jujube seed contained high amounts of higenamine and coclaurine, respectively (Fig. 5). A survey conducted by the Japan Kampo Medicines Manufacturers Association revealed that Sinomenium stem and rhizome, Phellodendron bark, and Jujube seed ranked among the top 60 most used crude drugs as pharmaceutical raw materials in Japan in 2020 [51]. Since the total usage of the top 60 crude drugs accounts for over 90% of the total usage, these crude drugs warrant greater attention from an anti-doping perspective. The content of coclaurine in Magnolia flower was the highest among the crude drugs investigated, measuring up to 1.95 µg/g in dry weight. Considering the average daily dosage of Magnolia flower (2.0 g) in Kampo medicines and the concentration of free higenamine (60.0 ng/mL) detected in the mice’s urine after the oral administration of coclaurine (310 µg), it is unlikely that coclaurine would cause a doping violation. However, since drug metabolism varies among species and individuals, athletes should exercise caution when taking crude drugs containing coclaurine or Kampo medicines made from such crude drugs.

## Conclusion

Currently, coclaurine is not listed in WADA’s International Standard Prohibited List. However, crude drugs containing coclaurine should be considered as a potential banned substance for athletes, just like those containing higenamine, because our results revealed that coclaurine is metabolized into higenamine. In this study, 14 crude drugs, including food ingredients, were screened to contain higenamine and/or coclaurine from 128 Kampo extract products by immunoassays, followed by LC–MS/MS analysis. Six crude drugs (Magnolia bark, Japanese Zanthoxylum peel, Jujube seed, Magnolia flower, Cimicifuga rhizome, and Coptis rhizome), and nine crude drugs (Cinnamon bark, Magnolia bark, Euodia fruit, Asiasarum root, Japanese Zanthoxylum peel, Cimicifuga rhizome, Jujube, Processed Aconite root, and Phellodendron bark) were newly found to contain higenamine and coclaurine, respectively. Considering the species and individual differences in drug metabolism, Kampo medicines containing these crude drugs should be administered carefully with caution by the athlete, regardless of the content of higenamine and coclaurine. Our results suggested that the intake of coclaurine, which is not classified as a banned substance, may lead to doping violations. These findings question the justification for the exclusive inclusion of higenamine in the WADA’s International Standard Prohibited List.

This study presents a method for screening crude drugs containing target compounds using Kampo extract products as a compound library. The approach can also be extended to the analysis of dietary supplements and related products. Consequently, it is expected to contribute significantly to the prevention of unintentional doping by athletes.

## Supplementary Information

Below is the link to the electronic supplementary material.

Supplementary file1 (DOCX 3399 KB)

## Data Availability

The data supporting the findings of this study are available from the corresponding author upon request.

## Abbreviations

AAF: Adverse analytical finding
CR: Cross-reactivity
CYP: Cytochrome P450
ELISA: Enzyme-linked immunosorbent assay
GC–MS: Gas chromatography–mass spectrometry
HLM: Human liver microsomes
icELISA: Indirect competitive enzyme-linked immunosorbent assay
LC–MS/MS: Liquid chromatography–tandem mass spectrometry
LFA: Lateral flow immunoassay
MAb: Monoclonal antibody
OVA: Ovalbumin
PBS: Phosphate-buffered saline
PBS-sm: PBS containing 5% skimmed milk
PBS-t: PBS containing 0.05% Tween-20; UDPGA, uridine 5′-diphosphoglucuronic acid
UGT: UDP-glucuronosyltransferase
UPLC–MS/MS: Ultra-performance liquid chromatography–tandem mass spectrometry
WADA: World Anti-Doping Agency
XIC: Extracted ion chromatogram
