# Supplementary material for: Metabolism of coclaurine into the WADA-banned substance higenamine: a doping-relevant analytical evaluation of Kampo extracts
Source: J Nat Med. 2025 Aug 2;79(5):1140–53. doi: 10.1007/s11418-025-01940-4 (PMC13144235; doi:10.1007/s11418-025-01940-4)
Supplement: Supplementary file 1 — Supplementary file1 (DOCX 3399 KB) [file 11418_2025_1940_MOESM1_ESM.docx]

**[Supplementary material]**

**Metabolism of coclaurine into the WADA-banned substance higenamine: A doping-relevant analytical evaluation of Kampo extracts**

Seiichi Sakamoto^1,*^, Kouta Osaki^1^, Hiroko Abe^2^, Yorie Tayama^1^, Akito Tsuruta^3^, Poomraphie Nuntawong^1^, Satoru Koyanagi^3^, Varalee Yodsurang^4,5^, Satoshi Morimoto^1^

**Affiliation**

^1^ Department of Pharmacognosy, Graduate School of Pharmaceutical Sciences, Kyushu University, 3-1-1 Maidashi, Higashi-ku, Fukuoka 812-8582, Japan

^2^ Biodesign Inc., 3-25-15 Nishi Ikebukuro, Toshima, Tokyo 171-0021, Japan

^3^ Department of Pharmaceutics, Graduate School of Pharmaceutical Sciences, Kyushu University, 3-1-1 Maidashi, Higashi-ku, Fukuoka 812-8582, Japan

^4^ Department of Pharmacology and Physiology, Faculty of Pharmaceutical Sciences, Chulalongkorn University, Bangkok 10330, Thailand

^5^ Center of Excellence in Preclinical Toxicity and Efficacy Assessment of Medicines and Chemicals, Chulalongkorn University, Bangkok 10330, Thailand

**Correspondence**

Seiichi Sakamoto, Associate Professor

Department of Pharmacognosy, Graduate School of Pharmaceutical Sciences, Kyushu University; 3-1-1 Maidashi, Higashi-ku, Fukuoka 812-8582, Japan. E-mail: s.sakamoto@phar.kyushu-u.ac.jp

Phone: +81 92 642 6581 Fax: +81 92 642 6581

**Material and methods**

**Sample preparation of metabolite analysis of coclaurine in mice for LC–MS/MS analysis**

To remove proteins by precipitations in urine, acetonitrile (50 μL) containing diazepam-d5 at 50 ng/mL was added into urine (50 μL), and they were centrifuged at 12,000 rpm for 5 min at 25^o^C. The supernatant was collected and used as the sample for analysis. The samples were stored at 4℃ until use.

For feces samples, they were ground into powder using a mortar and pestle after lyophilization. Subsequently, powdered feces were weighed (300 mg), and metabolites were extracted with methanol (700 µL) by means of sonication for 30 min. After centrifugation at 15,000 rpm for 5 min at 25^o^C, the supernatant was collected. This step was repeated twice more, and the collected supernatant was then evaporated under nitrogen gas. Methanol (100 µL) were added to the residue, and they were treated with acetonitrile containing diazepam-d5 in the same way as urine sample. Finally, the supernatant was mixed with same volume of DW, and used as the sample for analysis.　Both urinary and feces samples were kept at 4^o^C until use.

**Sample preparations of Kampo extract products for immunological assay**

To the ground Kampo extract products (2.5 g), distilled water (DW, 30 mL) was added, and sonicated for 30 min. After centrifugation at 12,000 rpm for 30 min at room temperature, supernatant was applied to the Diaion HP-20 (Sigma-Aldrich, MO, USA) resin equilibrated with DW. After washing the column with DW, bound compounds were eluted with methanol (20 mL). Eluates were then evaporated, and residues were dissolved in methanol (150 μL), which were diluted with DW to adjust the final concentration of methanol at 5% (v/v). After centrifugation at 15,000 rpm for 10 min at 4^o^C, supernatant was used as samples for further immunological screening.

**Sample preparations of candidate crude drugs for LC–MS/MS analysis**

The candidate crude drugs that were not provided as powder, were ground using an electric mill and passed through a stainless steel sieve (300 µm). To crude drugs powder (1.0 g), methanol (10 mL) was added, and sonicated for 20 min. After centrifugation at 12,000 rpm for 5 min at 4 ^o^C, the supernatant was collected. This step was repeated, and the mixture of the two supernatants were used as the extract. The extract was concentrated 5-fold by the nitrogen as needed. To the extract/concentrated extract (200 µL), DW (800 µL) was added and vortexed. After centrifugation at 12,000 rpm for 5 min at 4 ^o^C, diazepam-d5 (1 µL, 10 µg/mL) was added to the supernatant (500 µL) after filtration through Cosmonice Filter W (4 mm, 0.45 μm, Nacalai Tesque, Kyoto, Japan) and used as the sample for detection and determination of higenamine and coclaurine by LC–MS/MS analysis.

As for determination, standard solutions of higenamine and coclaurine were prepared in 20% methanol at ten difference concentrations ranging from 1 to 1000 ng/mL; 1, 5, 10, 25, 50, 100, 250, 500, 750, and 1000 ng/mL. Extracted ion chromatogram (XIC) was used to calculate peak areas. Diazepam-d5 was added to the standard solution at final concentration of 20 ng/mL as an internal standard.

**Table S1** Chromatographic gradient by LC–MS/MS analysis for the detection of (*S*)-coclaurine metabolites in mice urine and feces with gradient system of (A) and higenamine and coclaurine in crude drugs with gradient system of (B) and (C).

**(B)**

**(A)**

| Time (min) | Solvent A (%) | Solvent B (%) |
| --- | --- | --- |
| 0 | 100 | 0 |
| 6 | 90 | 10 |
| 18 | 0 | 100 |
| 24 | 0 | 100 |
| 24.01 | 100 | 0 |
| 35 | 100 | 0 |

| Time (min) | Solvent A (%) | Solvent B (%) |
| --- | --- | --- |
| 0 | 100 | 0 |
| 4 | 80 | 20 |
| 5 | 0 | 100 |
| 10 | 0 | 100 |
| 10.01 | 100 | 0 |
| 15 | 100 | 0 |

**(C)**

| Time (min) | Solvent A (%) | Solvent B (%) |
| --- | --- | --- |
| 0 | 100 | 0 |
| 4 | 80 | 20 |
| 7 | 0 | 100 |
| 10 | 0 | 100 |
| 10.01 | 100 | 0 |
| 15 | 100 | 0 |

Among 36 crude drug samples, 29 samples were analyzed using ZORBAX Eclipse Plus C18 Rapid Resolution HT column with the gradient system of (B), while 7 samples were analyzed using CORTECS T3 column with the gradient system of (C) to obtain clear data. The mobile phase consisted of 0.1% (v/v) formic acid (solvent A) and acetonitrile (solvent B) with flow rate at 0.25 mL/min.

**Table S2** Definition of 18 crude drugs screened by LFA and icELISA.

| Candidate crude drug | Definition |
| --- | --- |
| Immature orange | The immature fruit or the fruit cut crosswise of *Citrus aurantium* Linné var. *daidai* Makino, *Citrus aurantium* Linné or *Citrus natsudaidai* Hayata (Rutaceae). |
| Cinnamon bark | The bark of the trunk of *Cinnamomum cassia* J. Presl (Lauraceae), or such bark from which a part of the periderm has been removed. |
| Magnolia bark | The bark of the trunk of *Magnolia obovata* Thunberg (*Magnolia hypoleuca* Siebold et Zuccarini), Magnolia officinalis Rehder et Wilson or *Magnolia officinalis* Rehder et Wilson var. *biloba* Rehder et Wilson (Magnoliaceae). |
| Euodia fruit | The fruit of *Euodia officinalis* Dode (*Evodia officinalis* Dode), *Euodia bodinieri* Dode (*Evodia bodinieri* Dode) or *Euodia ruticarpa* Hooker filius et Thomson (*Evodia rutaecarpa* Bentham) (Rutaceae). |
| Asiasarum root | The root and rhizome of *Asiasarum heterotropoides* F. Maekawa var. *mandshuricum* F. Maekawa or *Asiasarum sieboldii* F. Maekawa (Aristolochiaceae) |
| Japanese Zanthoxylum peel | The pericarps of the ripe fruit of *Zanthoxylum piperitum* De Candolle (Rutaceae), from which the seeds separated from the pericarps have been mostly removed. |
| Jujube seed | The seed of *Zizyphus jujuba* Miller var. *spinosa* Hu ex H.F. Chou (Rhamnaceae) |
| Magnolia flower | The flower bud of *Magnolia biondii* Pampanini, *Magnolia heptapeta* Dandy (*Magnolia denudata* Desrousseaux), *Magnolia sprengeri* Pampanini, *Magnolia salicifolia* Maximowicz, or *Magnolia kobus* De Candolle (Magnoliaceae). |
| Cimicifuga rhizome | The rhizome of *Cimicifuga dahurica* Maximowicz, *Cimicifuga heracleifolia* Komarov, *Cimicifuga foetida* Linné or *Cimicifuga simplex* Turczaninow (Ranunculaceae). |
| Jujube | The fruit of *Zizyphus jujuba* Miller var. *inermis* Rehder (Rhamnaceae). |
| Clove | The flowering bud of *Syzygium aromaticum* Merrill et Perry (*Eugenia caryophyllata* Thunberg) (Myrtaceae). |
| Citrus unshiu peel | The pericarp of the ripe fruit of *Citrus unshiu* Marcowicz or *Citrus reticulata* Blanco (Rutaceae). |
| Processed Aconite root | The tuberous root of *Aconitum carmichaeli* Debeaux or *Aconitum japonicum* Thunberg (Ranunculaceae) prepared by autoclaving. |
| Sinomenium stem and rhizome | The climbing stem and rhizome of *Sinomenium acutum* Rehder et Wilson (Menispermaceae), usually cut transversely. |
| Phellodendron bark | The bark of *Phellodendron* *amurense* Ruprecht or *Phellodendron chinense* Schneider (Rutaceae), from which the periderm has been removed. |
| Coptis rhizome | The rhizome of *Coptis japonica* Makino, *Coptis chinensis* Franchet, *Coptis deltoidei* C.Y. Cheng et Hsiao or *Coptis teeta* Wallich (Ranunculaceae), from which the roots have been removed practically. |
| Alpinia officinarum rhizome | The rhizome of *Alpinia officinarum* Hance (Zingiberaceae). |
| Nelumbo seed | The seed of *Nelumbo nucifera* Gaertner (Nymphaeaceae), usually with the endocarp, sometime being removed the embryo. |

**Table S3** Summary of the detection of higenamine and coclaurine in candidate crude drugs by LC–MS/MS analysis.

| Candidate crude drug | Group A | | Group B | |
| --- | --- | --- | --- | --- |
|  | higenamine | coclaurine | higenamine | coclaurine |
| Immature orange | n.d. | n.d. | n.d. | n.d. |
| Cinnamon bark | [C_16_H_17_NO_3_ + H]^+^ = 272.1262  [C_16_H_15_O_3_]^+^ = 255.1006  [C_16_H_13_O_2_]^+^ = 237.0917  [C_10_H_9_O_2_]^+^ = 161.0599  [C_7_H_7_O]^+^ = 107.0492 | [C_17_H_19_NO_3_ + H]^+^ = 286.1443  [C_17_H_17_O_3_]^+^ = 269.1175  [C_16_H_13_O_2_]^+^ = 237.0915  [C_11_H_11_O_2_]^+^ = 175.0759  [C_7_H_7_O]^+^ = 107.0494 | [C_16_H_17_NO_3_ + H]^+^ = 272.1318  [C_16_H_15_O_3_]^+^ = 255.0966  [C_16_H_13_O_2_]^+^ = 237.0910  [C_10_H_9_O_2_]^+^ = 161.0609  [C_7_H_7_O]^+^ = 107.0489 | [C_17_H_19_NO_3_ + H]^+^ = 286.1445  [C_17_H_17_O_3_]^+^ = 269.1186  [C_16_H_13_O_2_]^+^ = 237.0918  [C_11_H_11_O_2_]^+^ = 175.0751  [C_7_H_7_O]^+^ = 107.0491 |
| Magnolia bark | [C_16_H_17_NO_3_ + H]^+^ = 272.1282  [C_16_H_15_O_3_]^+^ = 255.1006  [C_16_H_13_O_2_]^+^ = 237.0894  [C_10_H_9_O_2_]^+^ = 161.0597  [C_7_H_7_O]^+^ = 107.0493 | [C_17_H_19_NO_3_ + H]^+^ = 286.1436  [C_17_H_17_O_3_]^+^ = 269.1166  [C_16_H_13_O_2_]^+^ = 237.0909  [C_11_H_11_O_2_]^+^ = 175.0754  [C_7_H_7_O]^+^ = 107.0492 | [C_16_H_17_NO_3_ + H]^+^ = 272.1280  [C_16_H_15_O_3_]^+^ = 255.1023  [C_16_H_13_O_2_]^+^ = 237.0898  [C_10_H_9_O_2_]^+^ = 161.0599  [C_7_H_7_O]^+^ = 107.0495 | [C_17_H_19_NO_3_ + H]^+^ = 286.1444  [C_17_H_17_O_3_]^+^ = 269.1176  [C_16_H_13_O_2_]^+^ = 237.0919  [C_11_H_11_O_2_]^+^ = 175.0760  [C_7_H_7_O]^+^ = 107.0494 |
| Euodia fruit | [C_16_H_17_NO_3_ + H]^+^ = 272.1275  [C_16_H_15_O_3_]^+^ = 255.1019  [C_16_H_13_O_2_]^+^ = 237.0918  [C_10_H_9_O_2_]^+^ = 161.0601  [C_7_H_7_O]^+^ = 107.0493 | [C_17_H_19_NO_3_ + H]^+^ = 286.1423  [C_17_H_17_O_3_]^+^ = 269.1188  [C_16_H_13_O_2_]^+^ = 237.0919  [C_11_H_11_O_2_]^+^ = 175.0751  [C_7_H_7_O]^+^ = 107.0491 | [C_16_H_17_NO_3_ + H]^+^ = 272.1299  [C_16_H_15_O_3_]^+^ = 255.1025  [C_16_H_13_O_2_]^+^ = 237.0903  [C_10_H_9_O_2_]^+^ = 161.0606  [C_7_H_7_O]^+^ = 107.0494 | [C_17_H_19_NO_3_ + H]^+^ = 286.1445  [C_17_H_17_O_3_]^+^ = 269.1183  [C_16_H_13_O_2_]^+^ = 237.0908  [C_11_H_11_O_2_]^+^ = 175.0756  [C_7_H_7_O]^+^ = 107.0496 |
| Asiasarum root | [C_16_H_17_NO_3_ + H]^+^ = 272.1280  [C_16_H_15_O_3_]^+^ = 255.1028  [C_16_H_13_O_2_]^+^ = 237.0905  [C_10_H_9_O_2_]^+^ = 161.0603  [C_7_H_7_O]^+^ = 107.0492 | [C_17_H_19_NO_3_ + H]^+^ = 286.1436  [C_17_H_17_O_3_]^+^ = 269.1186  [C_16_H_13_O_2_]^+^ = 237.0895  [C_11_H_11_O_2_]^+^ = 175.0759  [C_7_H_7_O]^+^ = 107.0493 | [C_16_H_17_NO_3_ + H]^+^ = 272.1278  [C_16_H_15_O_3_]^+^ = 255.1024  [C_16_H_13_O_2_]^+^ = 237.0909  [C_10_H_9_O_2_]^+^ = 161.0601  [C_7_H_7_O]^+^ = 107.0495 | [C_17_H_19_NO_3_ + H]^+^ = 286.1414  [C_17_H_17_O_3_]^+^ = 269.1165  [C_16_H_13_O_2_]^+^ = 237.0919  [C_11_H_11_O_2_]^+^ = 175.0752  [C_7_H_7_O]^+^ = 107.0493 |
| Japanese Zanthoxylum peel | [C_16_H_17_NO_3_ + H]^+^ = 272.1273  [C_16_H_15_O_3_]^+^ = 255.1015  [C_16_H_13_O_2_]^+^ = 237.0894  [C_10_H_9_O_2_]^+^ = 161.0585  [C_7_H_7_O]^+^ = 107.0491 | [C_17_H_19_NO_3_ + H]^+^ = 286.1447  [C_17_H_17_O_3_]^+^ = 269.1178  [C_16_H_13_O_2_]^+^ = 237.0909  [C_11_H_11_O_2_]^+^ = 175.0755  [C_7_H_7_O]^+^ = 107.0494 | [C_16_H_17_NO_3_ + H]^+^ = 272.1283  [C_16_H_15_O_3_]^+^ = 255.1030  [C_16_H_13_O_2_]^+^ = 237.0869  [C_10_H_9_O_2_]^+^ = 161.0582  [C_7_H_7_O]^+^ = 107.0488 | [C_17_H_19_NO_3_ + H]^+^ = 286.1431  [C_17_H_17_O_3_]^+^ = 269.1172  [C_16_H_13_O_2_]^+^ = 237.0908  [C_11_H_11_O_2_]^+^ = 175.0750  [C_7_H_7_O]^+^ = 107.0491 |
| Jujube seed | [C_16_H_17_NO_3_ + H]^+^ = 272.1275  [C_16_H_15_O_3_]^+^ = 255.1024  [C_10_H_9_O_2_]^+^ = 161.0582  [C_7_H_7_O]^+^ = 107.0490 | [C_17_H_19_NO_3_ + H]^+^ = 286.1438  [C_17_H_17_O_3_]^+^ = 269.1175  [C_16_H_13_O_2_]^+^ = 237.0912  [C_11_H_11_O_2_]^+^ = 175.0755  [C_7_H_7_O]^+^ = 107.0494 | [C_16_H_17_NO_3_ + H]^+^ = 272.1281  [C_16_H_15_O_3_]^+^ = 255.1040  [C_16_H_13_O_2_]^+^ = 237.0939  [C_10_H_9_O_2_]^+^ = 161.0597  [C_7_H_7_O]^+^ = 107.0483 | [C_17_H_19_NO_3_ + H]^+^ = 286.1442  [C_17_H_17_O_3_]^+^ = 269.1176  [C_16_H_13_O_2_]^+^ = 237.0915  [C_11_H_11_O_2_]^+^ = 175.0755  [C_7_H_7_O]^+^ = 107.0492 |
| Magnolia flower | [C_16_H_17_NO_3_ + H]^+^ = 272.1286  [C_16_H_15_O_3_]^+^ = 255.1021  [C_10_H_9_O_2_]^+^ = 161.0597  [C_7_H_7_O]^+^ = 107.0492 | [C_17_H_19_NO_3_ + H]^+^ = 286.1431  [C_17_H_17_O_3_]^+^ = 269.1164  [C_16_H_13_O_2_]^+^ = 237.0904  [C_11_H_11_O_2_]^+^ = 175.0750  [C_7_H_7_O]^+^ = 107.0489 | [C_16_H_17_NO_3_ + H]^+^ = 272.1286  [C_16_H_15_O_3_]^+^ = 255.1025  [C_16_H_13_O_2_]^+^ = 237.0914  [C_10_H_9_O_2_]^+^ = 161.0602  [C_7_H_7_O]^+^ = 107.0492 | [C_17_H_19_NO_3_ + H]^+^ = 286.1430  [C_17_H_17_O_3_]^+^ = 269.1167  [C_16_H_13_O_2_]^+^ = 237.0901  [C_11_H_11_O_2_]^+^ = 175.0747  [C_7_H_7_O]^+^ = 107.0494 |
| Cimicifuga rhizome | [C_16_H_17_NO_3_ + H]^+^ = 272.1268  [C_16_H_15_O_3_]^+^ = 255.1017  [C_16_H_13_O_2_]^+^ = 237.0869  [C_10_H_9_O_2_]^+^ = 161.0599  [C_7_H_7_O]^+^ = 107.0496 | [C_17_H_19_NO_3_ + H]^+^ = 286.1445  [C_17_H_17_O_3_]^+^ = 269.1161  [C_16_H_13_O_2_]^+^ = 237.0909  [C_11_H_11_O_2_]^+^ = 175.0777  [C_7_H_7_O]^+^ = 107.0493 | [C_16_H_17_NO_3_ + H]^+^ = 272.1294  [C_16_H_15_O_3_]^+^ = 255.1011  [C_10_H_9_O_2_]^+^ = 161.0598  [C_7_H_7_O]^+^ = 107.0491 | [C_17_H_19_NO_3_ + H]^+^ = 286.1440  [C_17_H_17_O_3_]^+^ = 269.1191  [C_16_H_13_O_2_]^+^ = 237.0993  [C_11_H_11_O_2_]^+^ = 175.0780  [C_7_H_7_O]^+^ = 107.0495 |
| Jujube | n.d. | [C_17_H_19_NO_3_ + H]^+^ = 286.1431  [C_17_H_17_O_3_]^+^ = 269.1176  [C_16_H_13_O_2_]^+^ = 237.0902  [C_11_H_11_O_2_]^+^ = 175.0754  [C_7_H_7_O]^+^ = 107.0491 | n.d. | [C_17_H_19_NO_3_ + H]^+^ = 286.1440  [C_17_H_17_O_3_]^+^ = 269.1171  [C_16_H_13_O_2_]^+^ = 237.0911  [C_11_H_11_O_2_]^+^ = 175.0758  [C_7_H_7_O]^+^ = 107.0493 |
| Clove | n.d. | n.d. | n.d. | n.d. |
| Citrus unshiu peel | n.d. | n.d. | n.d. | n.d. |

n.d. represents not detected.

| Processed Aconite root | [C_16_H_17_NO_3_ + H]^+^ = 272.1285  [C_16_H_15_O_3_]^+^ = 255.1019  [C_16_H_13_O_2_]^+^ = 237.0911  [C_10_H_9_O_2_]^+^ = 161.0600  [C_7_H_7_O]^+^ = 107.0491 | [C_17_H_19_NO_3_ + H]^+^ = 286.1442  [C_17_H_17_O_3_]^+^ = 269.1174  [C_16_H_13_O_2_]^+^ = 237.0919  [C_11_H_11_O_2_]^+^ = 175.0754  [C_7_H_7_O]^+^ = 107.0494 | [C_16_H_17_NO_3_ + H]^+^ = 272.1289  [C_16_H_15_O_3_]^+^ = 255.1012  [C_16_H_13_O_2_]^+^ = 237.0915  [C_10_H_9_O_2_]^+^ = 161.0601  [C_7_H_7_O]^+^ = 107.0491 | [C_17_H_19_NO_3_ + H]^+^ = 286.1439  [C_17_H_17_O_3_]^+^ = 269.1168  [C_16_H_13_O_2_]^+^ = 237.0920  [C_11_H_11_O_2_]^+^ = 175.0762  [C_7_H_7_O]^+^ = 107.0489 |
| --- | --- | --- | --- | --- |
| Sinomenium stem and rhizome | [C_16_H_17_NO_3_ + H]^+^ = 272.1280  [C_16_H_15_O_3_]^+^ = 255.1015  [C_16_H_13_O_2_]^+^ = 237.0907  [C_10_H_9_O_2_]^+^ = 161.0597  [C_7_H_7_O]^+^ = 107.0495 | [C_17_H_19_NO_3_ + H]^+^ = 286.1441  [C_17_H_17_O_3_]^+^ = 269.1175  [C_16_H_13_O_2_]^+^ = 237.0913  [C_11_H_11_O_2_]^+^ = 175.0755  [C_7_H_7_O]^+^ = 107.0492 | [C_16_H_17_NO_3_ + H]^+^ = 272.1275  [C_16_H_15_O_3_]^+^ = 255.1011  [C_16_H_13_O_2_]^+^ = 237.0905  [C_10_H_9_O_2_]^+^ = 161.0596  [C_7_H_7_O]^+^ = 107.0490 | [C_17_H_19_NO_3_ + H]^+^ = 286.1437  [C_17_H_17_O_3_]^+^ = 269.1170  [C_16_H_13_O_2_]^+^ = 237.0908  [C_11_H_11_O_2_]^+^ = 175.0754  [C_7_H_7_O]^+^ = 107.0490 |
| Phellodendron bark | [C_16_H_17_NO_3_ + H]^+^ = 272.1274  [C_16_H_15_O_3_]^+^ = 255.1020  [C_16_H_13_O_2_]^+^ = 237.0914  [C_10_H_9_O_2_]^+^ = 161.0592  [C_7_H_7_O]^+^ = 107.0492 | [C_17_H_19_NO_3_ + H]^+^ = 286.1461  [C_17_H_17_O_3_]^+^ = 269.1182  [C_16_H_13_O_2_]^+^ = 237.0915  [C_11_H_11_O_2_]^+^ = 175.0762  [C_7_H_7_O]^+^ = 107.0494 | [C_16_H_17_NO_3_ + H]^+^ = 272.1294  [C_16_H_15_O_3_]^+^ = 255.1023  [C_16_H_13_O_2_]^+^ = 237.0922  [C_10_H_9_O_2_]^+^ = 161.0597  [C_7_H_7_O]^+^ = 107.0494 | [C_17_H_19_NO_3_ + H]^+^ = 286.1428  [C_17_H_17_O_3_]^+^ = 269.1179  [C_16_H_13_O_2_]^+^ = 237.0909  [C_11_H_11_O_2_]^+^ = 175.0751  [C_7_H_7_O]^+^ = 107.0492 |
| Coptis rhizome | [C_16_H_17_NO_3_ + H]^+^ = 272.1271  [C_16_H_15_O_3_]^+^ = 255.1013  [C_16_H_13_O_2_]^+^ = 237.0912  [C_10_H_9_O_2_]^+^ = 161.0596  [C_7_H_7_O]^+^ = 107.0492 | n.d. | [C_16_H_17_NO_3_ + H]^+^ = 272.1266  [C_16_H_15_O_3_]^+^ = 255.1026  [C_16_H_13_O_2_]^+^ = 237.0927  [C_10_H_9_O_2_]^+^ = 161.0596  [C_7_H_7_O]^+^ = 107.0493 | n.d. |
| Alpinia officinarum rhizome | n.d. | n.d. | n.d. | n.d. |
| Nelumbo seed | [C_16_H_17_NO_3_ + H]^+^ = 272.1276  [C_16_H_15_O_3_]^+^ = 255.1013  [C_16_H_13_O_2_]^+^ = 237.0878  [C_10_H_9_O_2_]^+^ = 161.0595  [C_7_H_7_O]^+^ = 107.0490 | [C_17_H_19_NO_3_ + H]^+^ = 286.1456  [C_17_H_17_O_3_]^+^ = 269.1155  [C_16_H_13_O_2_]^+^ = 237.0907  [C_11_H_11_O_2_]^+^ = 175.0764  [C_7_H_7_O]^+^ = 107.0481 | [C_16_H_17_NO_3_ + H]^+^ = 272.1290  [C_16_H_15_O_3_]^+^ = 255.1002  [C_16_H_13_O_2_]^+^ = 237.0921  [C_10_H_9_O_2_]^+^ = 161.0600  [C_7_H_7_O]^+^ = 107.0497 | [C_17_H_19_NO_3_ + H]^+^ = 286.1442  [C_17_H_17_O_3_]^+^ = 269.1175  [C_16_H_13_O_2_]^+^ = 237.0915  [C_11_H_11_O_2_]^+^ = 175.0764  [C_7_H_7_O]^+^ = 107.0494 |

n.d. represents not detected.

**
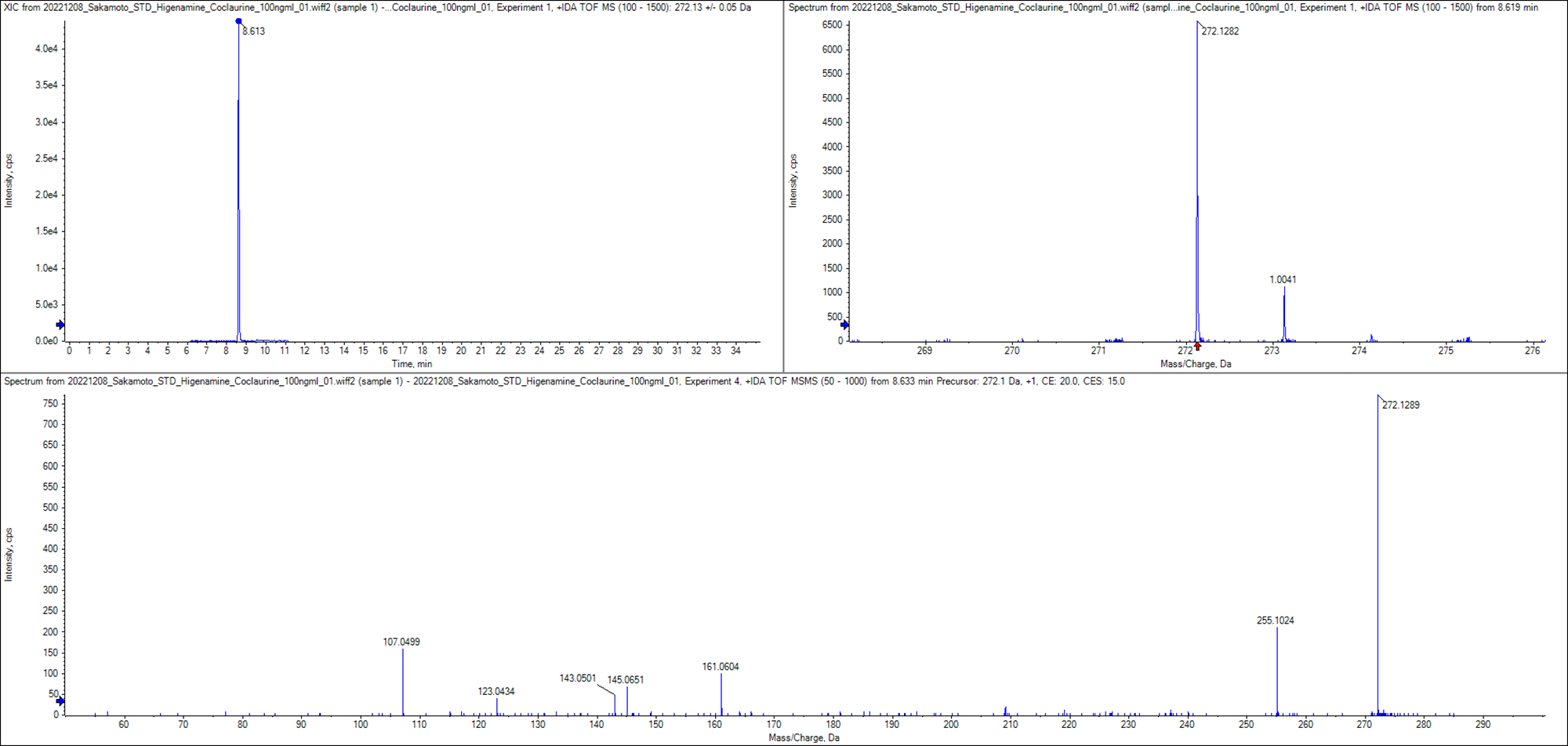
Figure S1** LC–MS/MS spectra of standard (A) higenamine and (B) coclaurine.

**(A)**

**
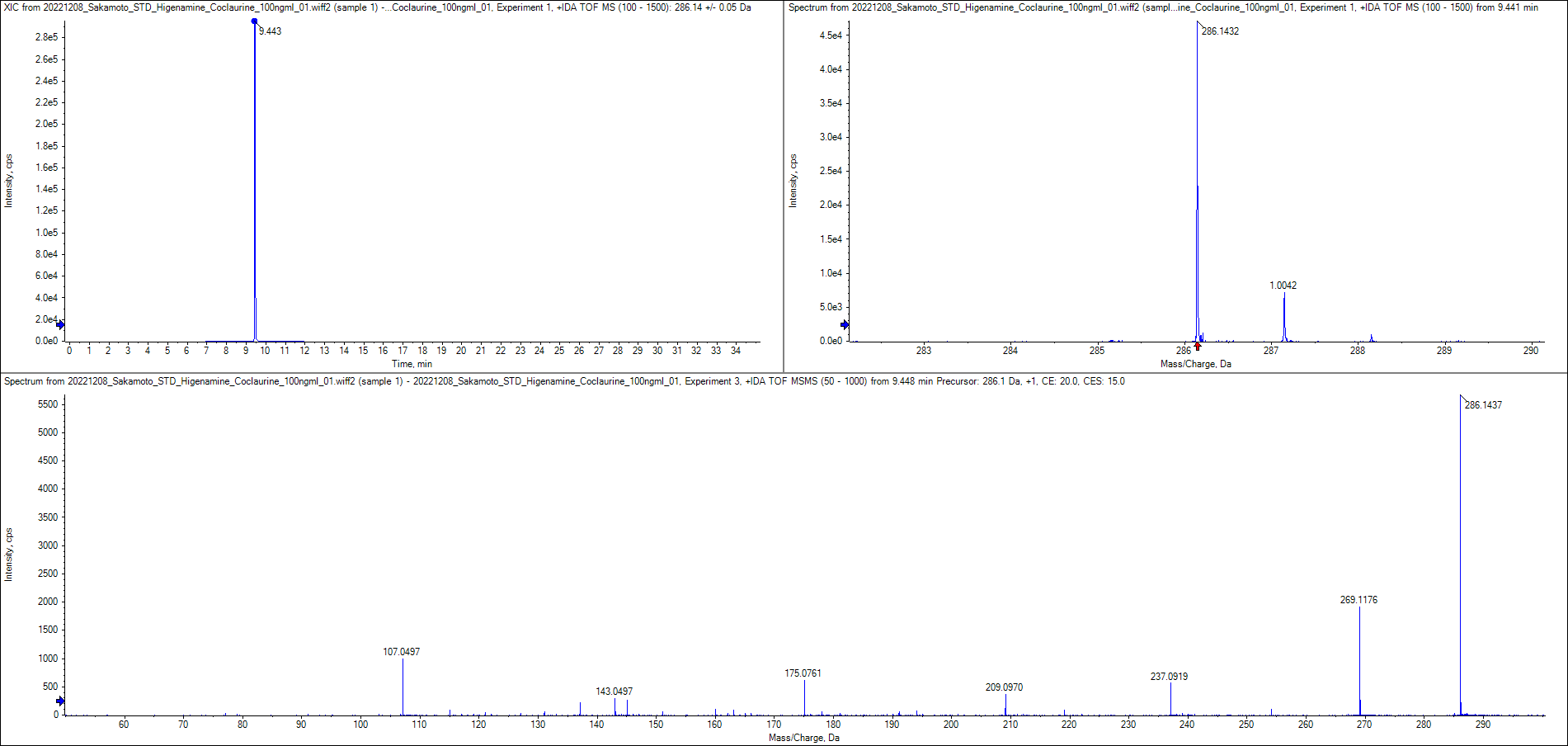
**

**(B)**

Upper left, upper right, and lower spectra indicate extracted ion chromatogram (XIC), MS, and MS/MS spectra, respectively.

XIC of higenamine and coclaurine was obtained at *m/z* 272.128 ± 0.005 and 286.144 ± 0.005, respectively.

**Figure S2** Detection example of (A) higenamine-glucuronide and (B) coclaurine-glucuronide in urine sample by LC–MS/MS analysis.

**
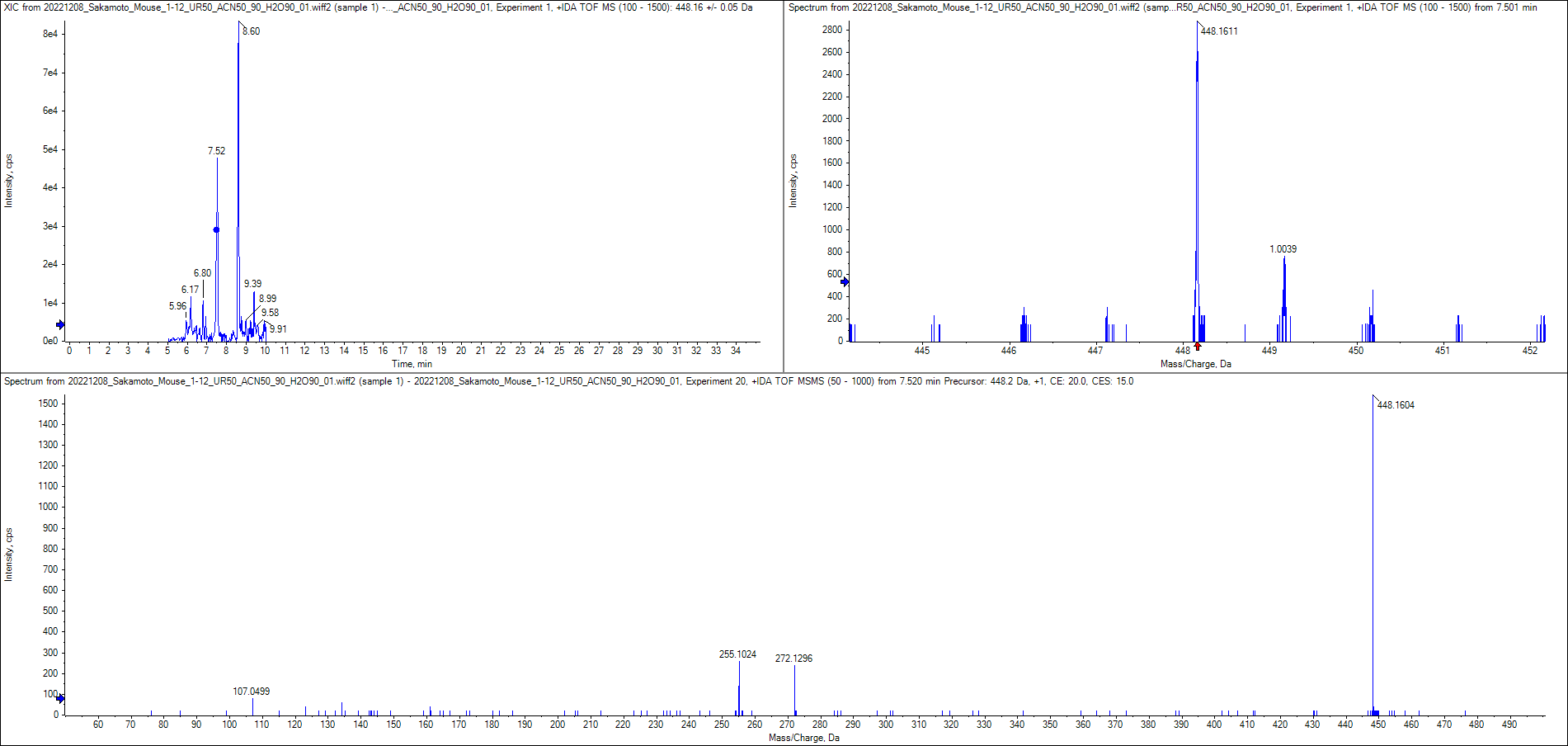
**

**(A)**

**(B)**

**
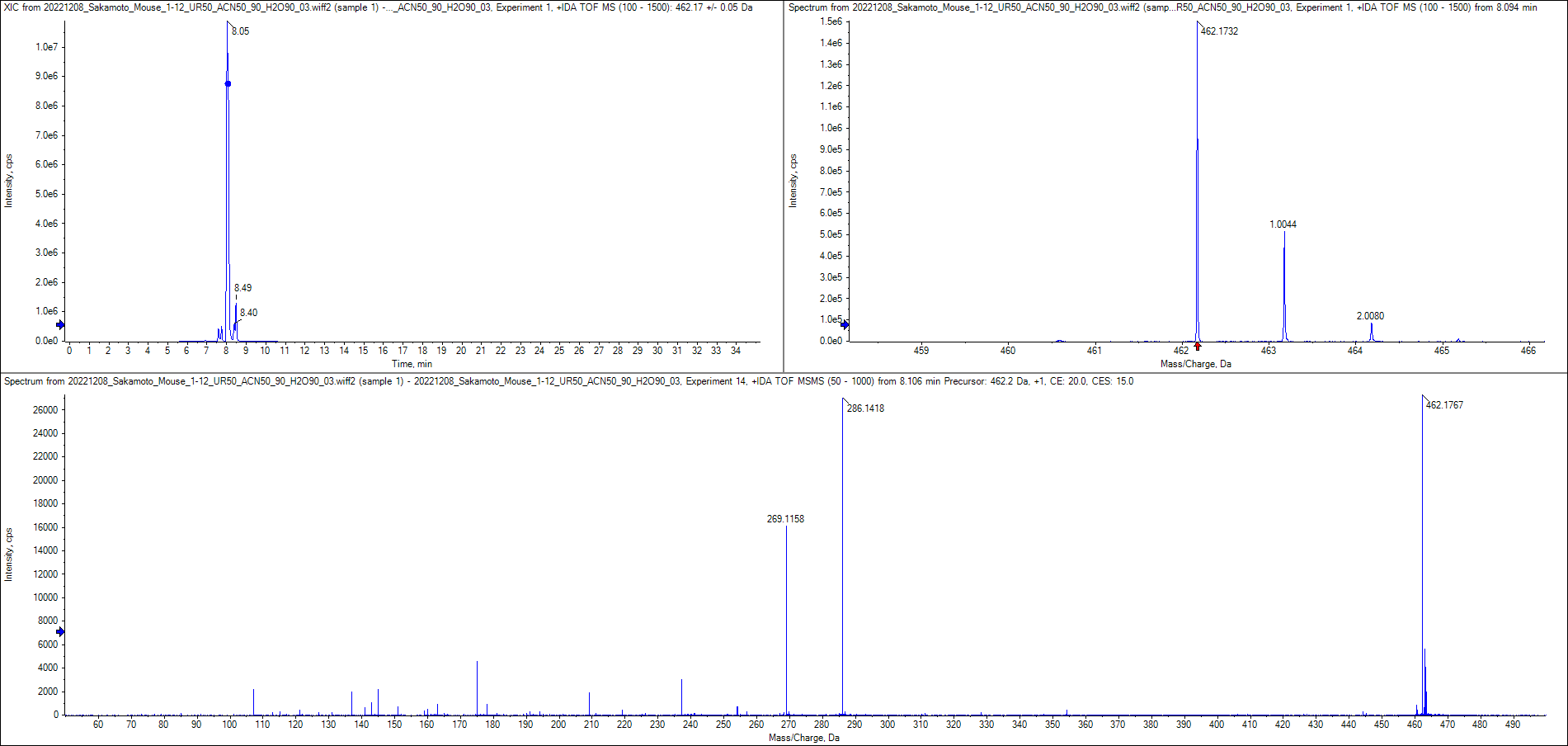
**

Upper left, upper right, and lower spectra indicate XIC, MS, and MS/MS spectra, respectively.

XIC of higenamine-glucuronide and coclaurine-glucuronide was obtained at *m/z* 448.16 ± 0.05 and 462.17 ± 0.05, respectively.

**Figure S3** Screening of crude drugs containing higenamine and/or coclaurine from 128 kinds of Kampo extract products by LFA using MAb E8.

**(A)**


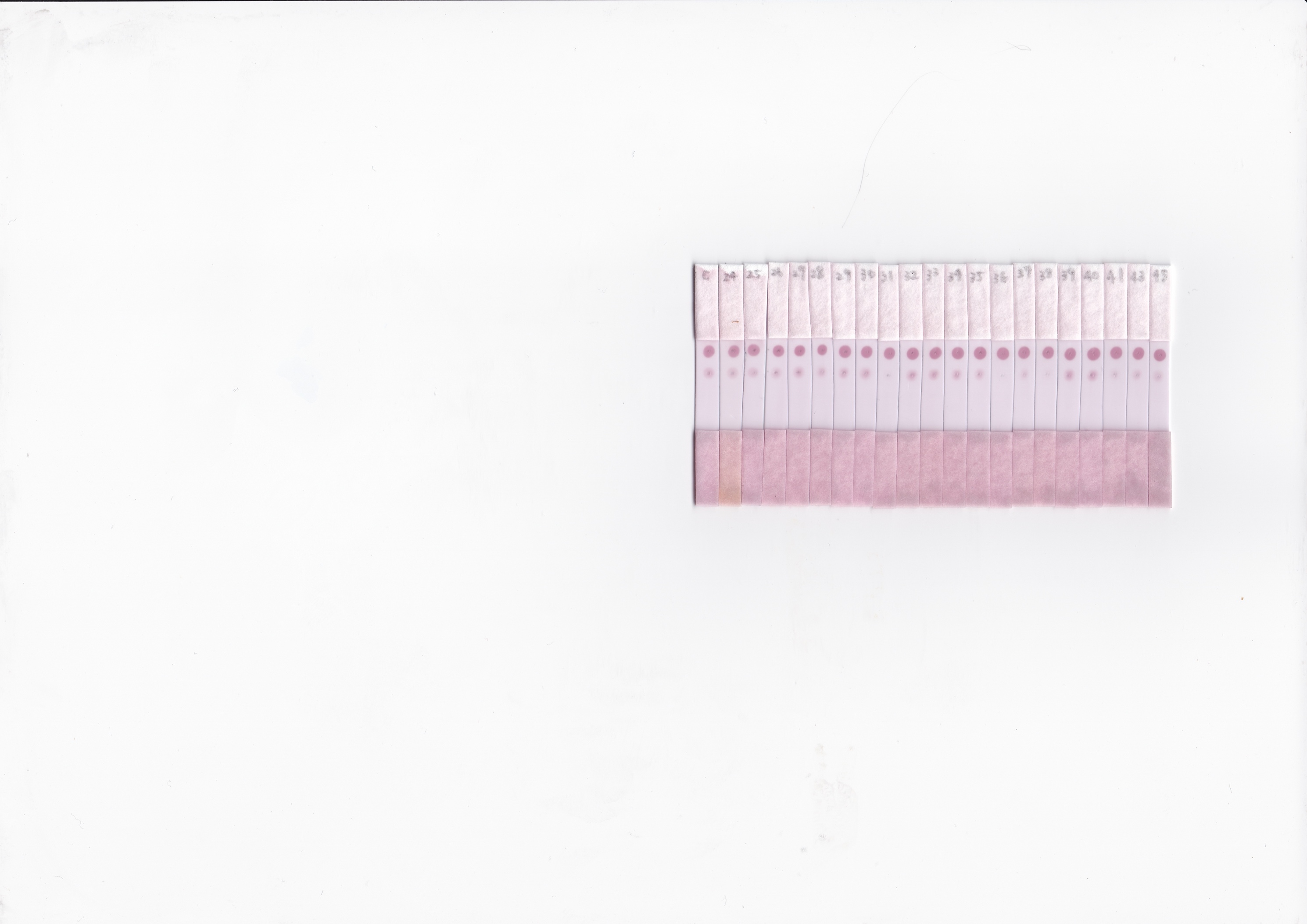

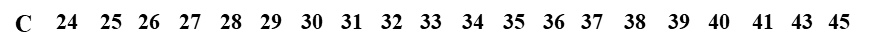


**++**

**++**

**++**

**+**

**+**

**+**

**+**


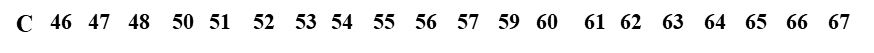

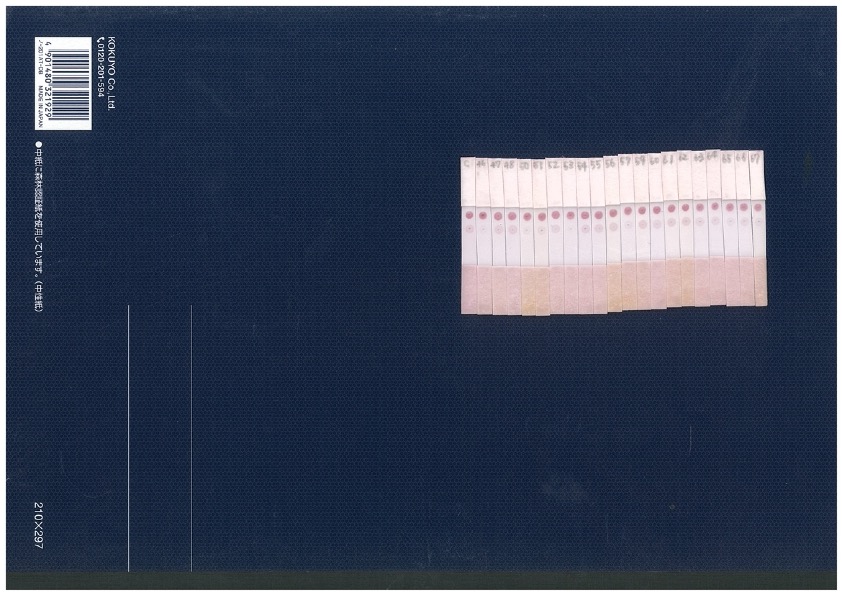


**(B)**

**+**

**++**

**+**

**+**

**+**

**++**

**++**


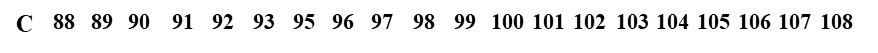

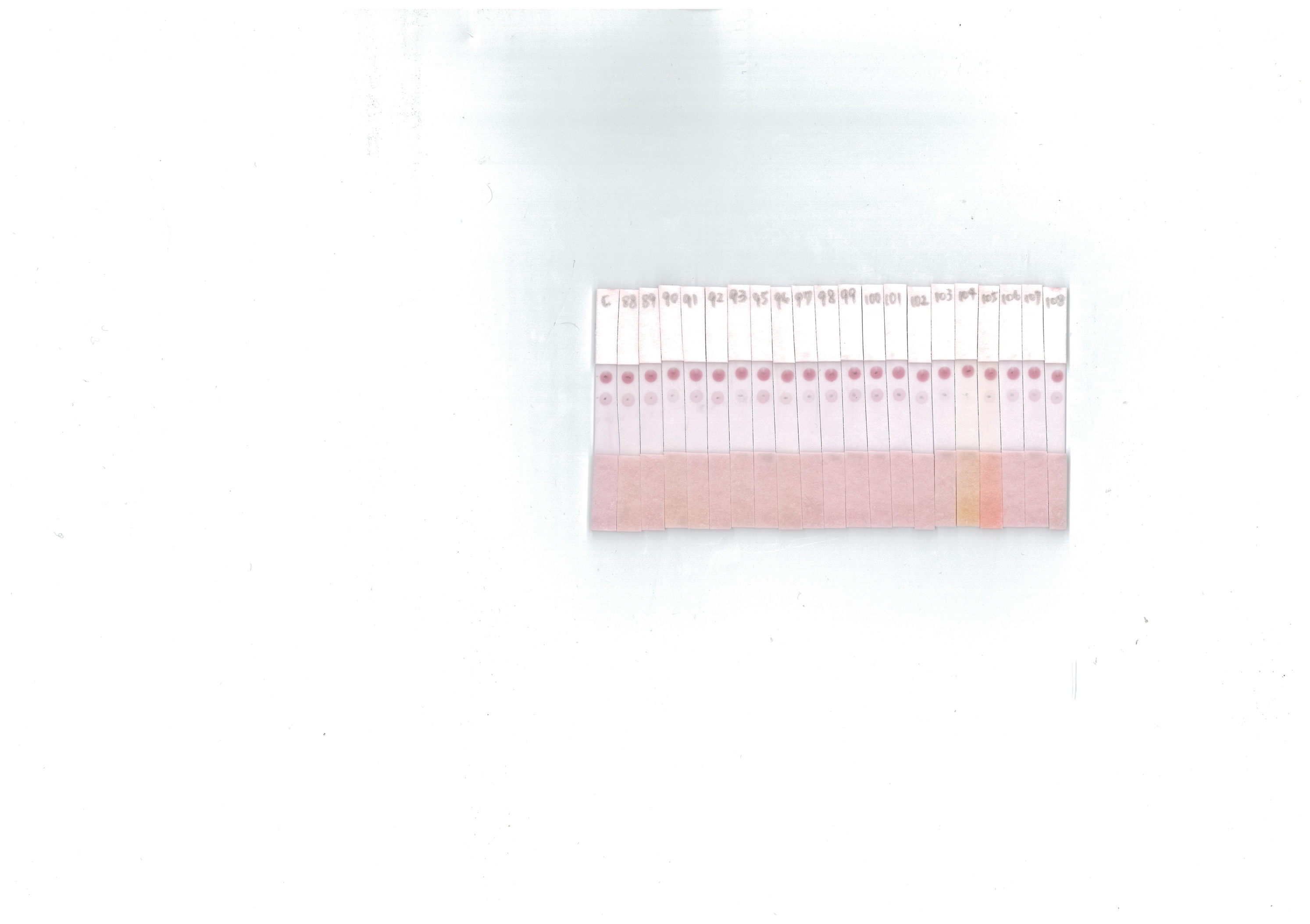

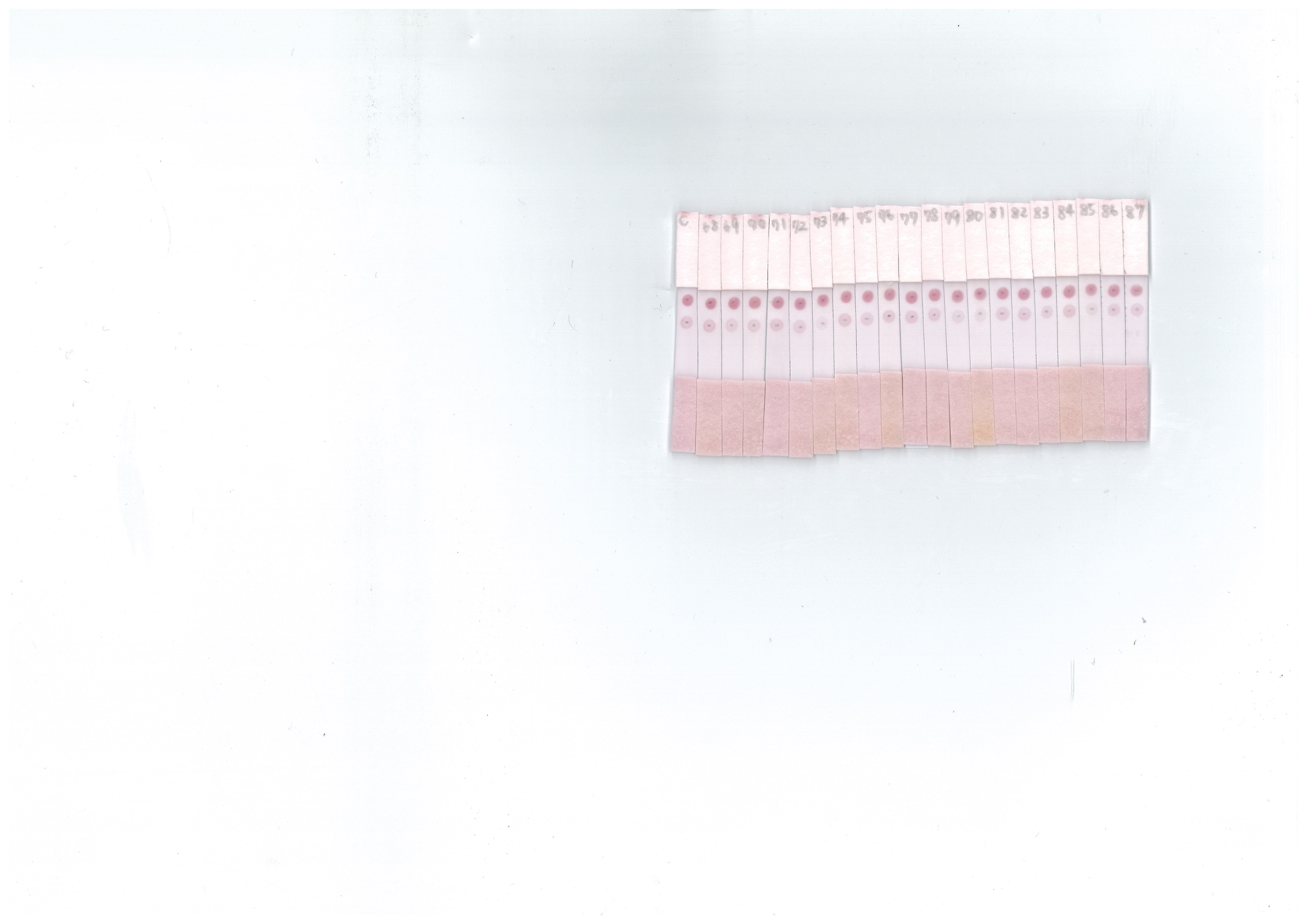

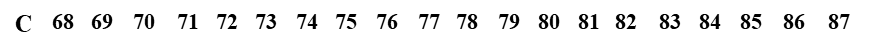


**(D)**

**(C)**

**+**

**+**

**+**

**+**

**++**

**++**

**++**

**+**

**+**

**+**

**+**


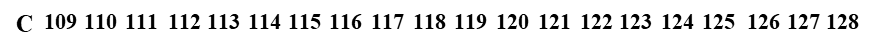

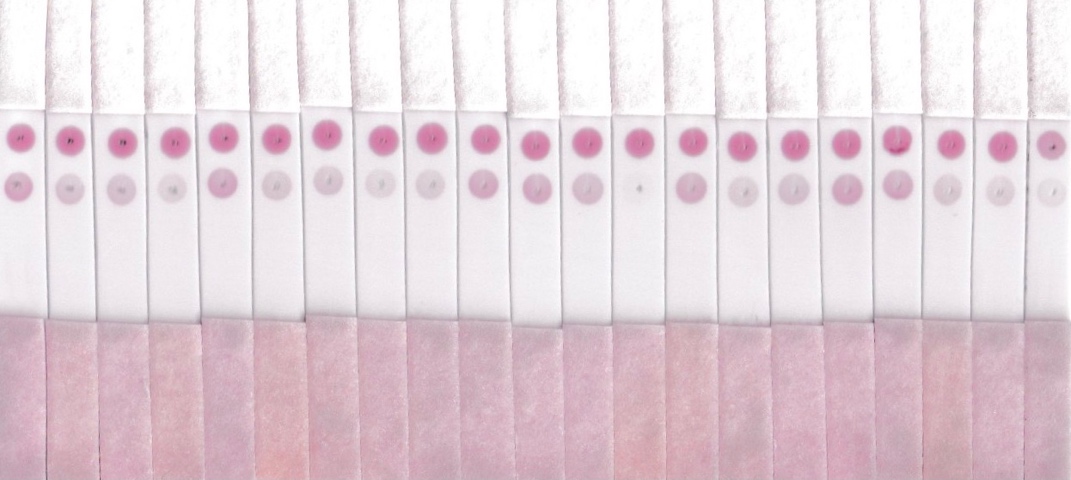


**(E)**

**+**

**++**

**(F)**


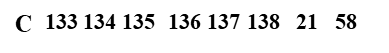

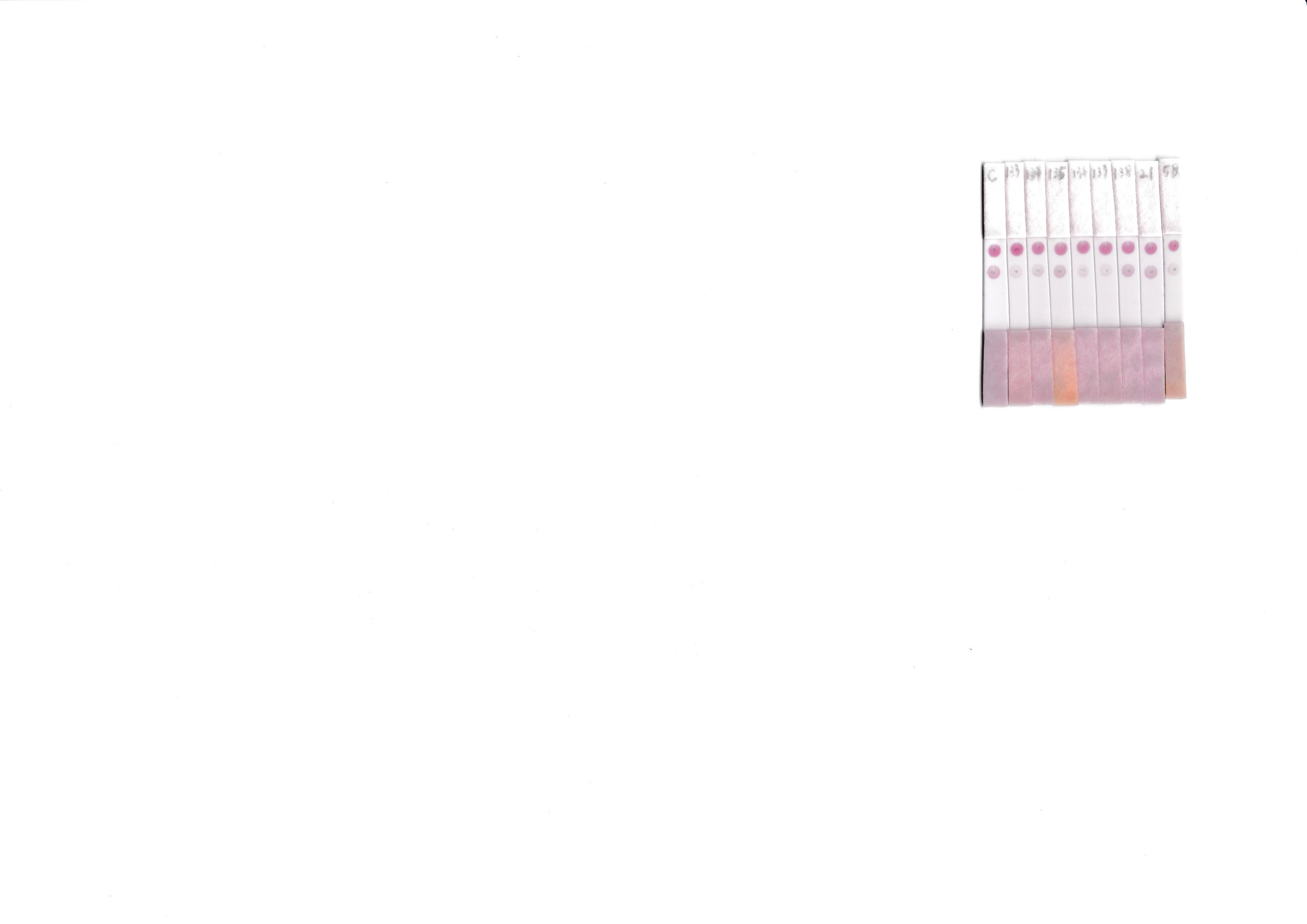


**+**

**+**

**+**

**+**

C indicates the control, where 5% (v/v) methanol was used, and the numbers on the strip test correspond to the product No. of TSUMURA assigned to 128 Kampo extract products; (A) 24－45, (B) 46－67 (except 58), (C) 68－87, (D) 88－108, (E) 109－128, and (F) 133－138, 21, and 58. The (++) and (+) indicate positive and weak positive, respectively.

**Figure S4** Screening of crude drugs containing higenamine and/or coclaurine from 128 kinds of Kampo extract products by icELISA using MAb E8.


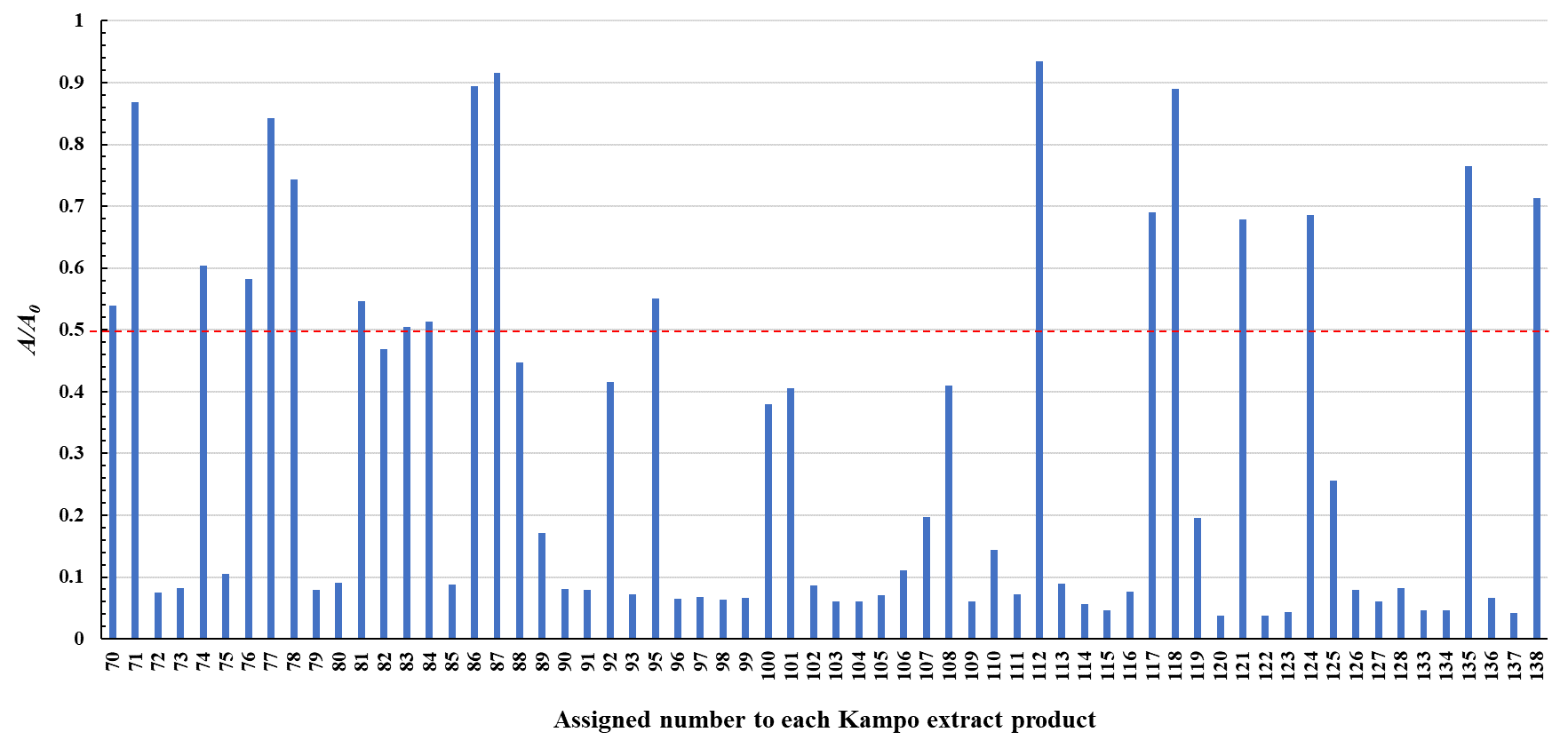


*A* and *A_0_* indicate the absorbance obtained from each sample of Kampo extract products and the negative control (5% (v/v) methanol), respectively. The numbers (70－138) of the X axis correspond to the product No. of TSUMURA assigned to 128 Kampo extract products.

**Figure S5** LC–MS/MS spectra of standard (A) higenamine and (B) coclaurine.

**(A)**

**
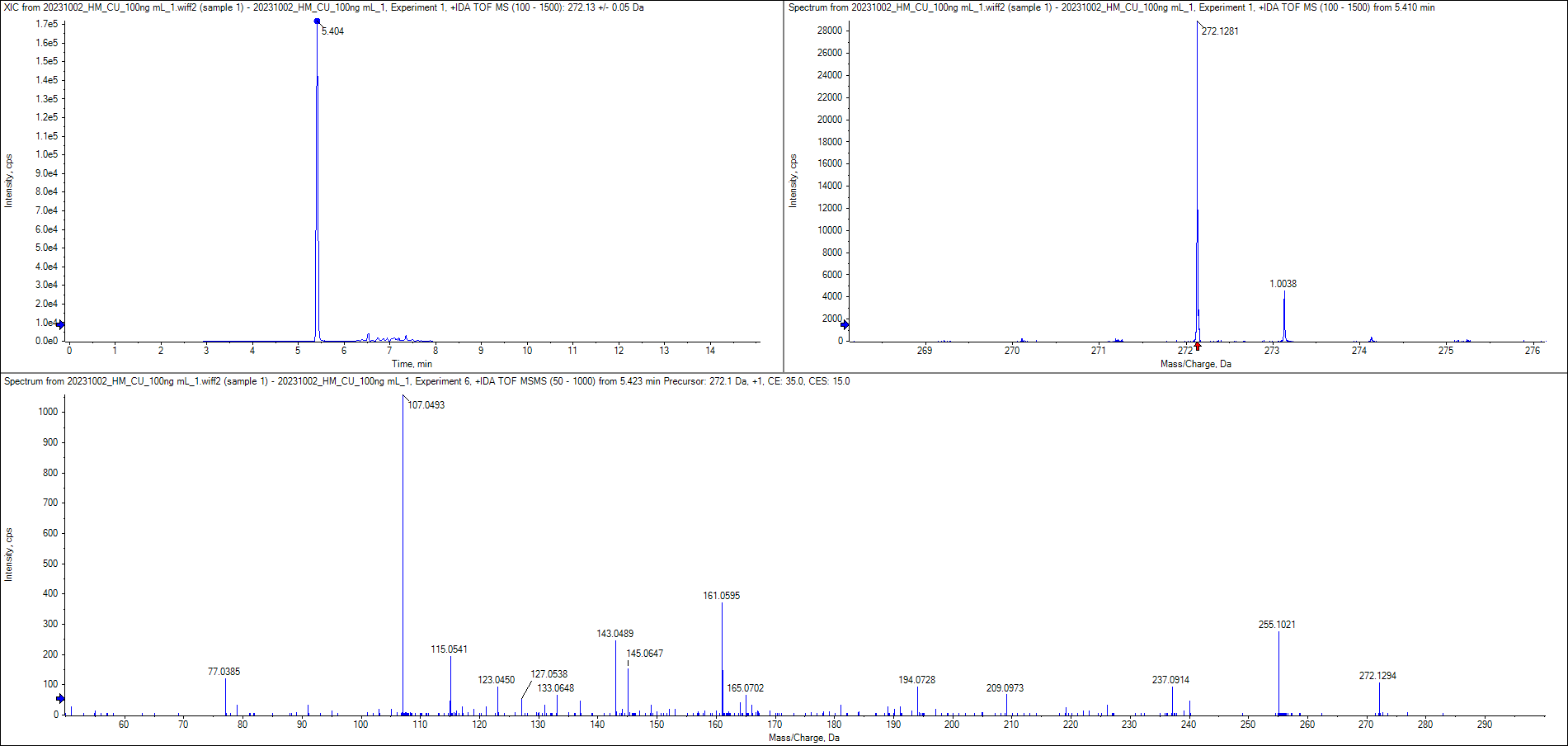
**

**(B)**

**
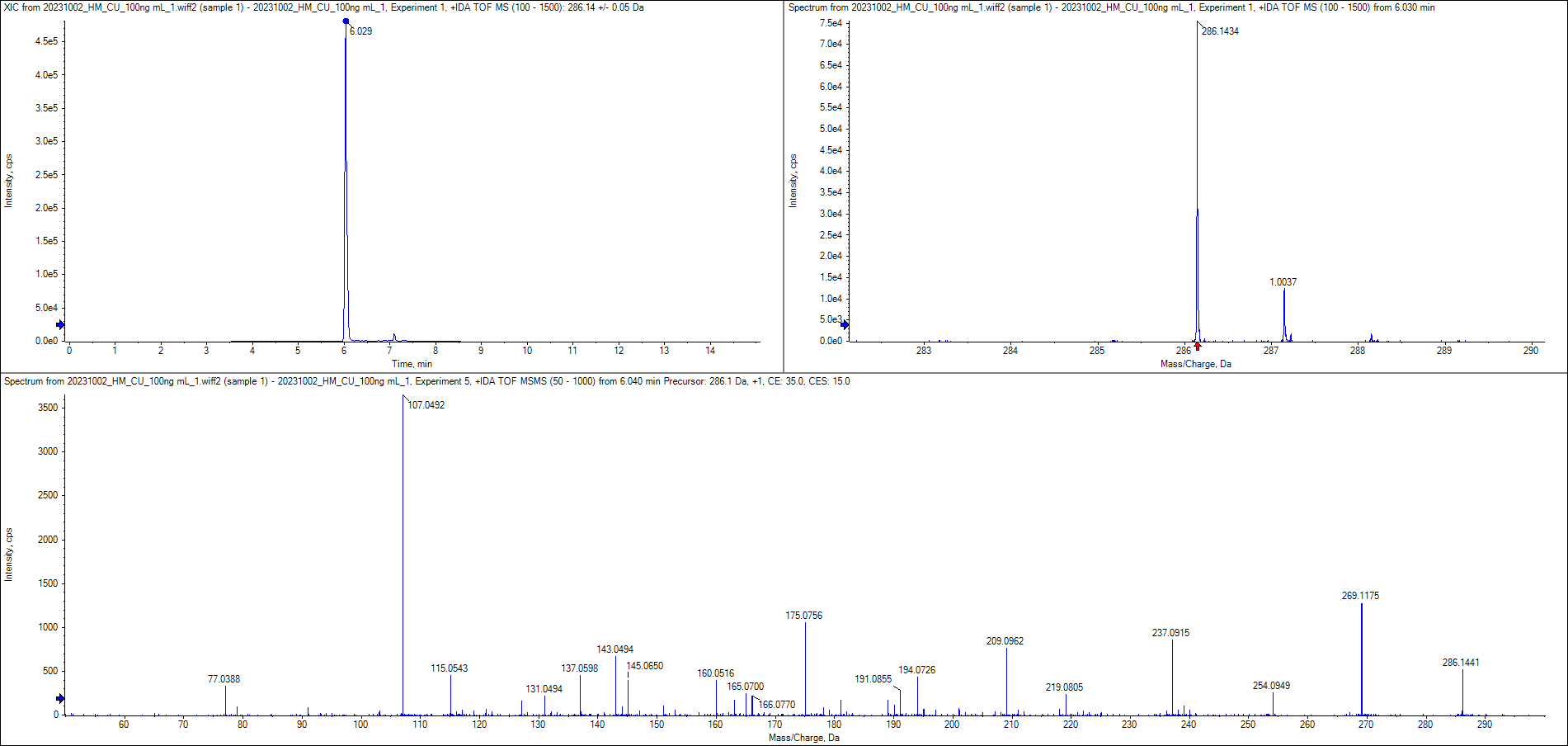
**

Upper left, upper right, and lower spectra indicate extracted ion chromatogram (XIC), MS, and MS/MS spectra, respectively.

XIC of higenamine and coclaurine was obtained at *m/z* 272.128 ± 0.005 and 286.144 ± 0.005, respectively.
